# Insights into Non-Antibiotic Alternative and Emerging Control Strategies for Chicken Coccidiosis

**DOI:** 10.3390/ani16020348

**Published:** 2026-01-22

**Authors:** Rochelle A. Flores, Paula Leona C. Fletcher, Kyu-Yeol Son, Wongi Min

**Affiliations:** 1 College of Veterinary Medicine & Institute of Animal Medicine, Gyeongsang National University, Jinju 52828, Republic of Korea; floresrochellea@gmail.com; 2 Department of Veterinary Paraclinical Sciences, College of Veterinary Medicine, University of the Philippines Los Baños, Los Baños 4031, Laguna, Philippines; cammayopaula@gmail.com; 3 R&D Division, CJ CheilJedang Corporation, CJ CheilJedang Center, 330 Donghoro, Junggolu, Seoul 04560, Republic of Korea; umyfriends@naver.com; 4 Hoxbio, Business Center, Gyeongsang National University, Jinju 52828, Republic of Korea

**Keywords:** chickens, *Eimeria*, innovative strategies, microbiome-modulating agents, natural products, nanodelivery systems, non-antibiotic control, phytochemicals, omics-guided technologies

## Abstract

Coccidiosis, caused by an obligate intracellular parasite of the genus *Eimeria*, is the most economically parasitic disease in poultry. Long-term reliance on synthetic anticoccidials and ionophores has accelerated the emergence of drug resistance and intensified the need for effective, residue-free alternatives. This narrative review synthesizes findings from peer-reviewed studies published between 1998 and 2025, summarizing advances in non-antibiotic control strategies encompassing five domains: (i) phytochemicals and botanicals, (ii) functional nutrition and mineral modulators, (iii) microbial and gut modulators, (iv) host-directed immunological and biotechnological approaches, and (v) precision and omics-guided biotherapeutic platforms. These approaches consistently reduce lesion severity, oocyst shedding, oxidative stress, and mortality while improving growth parameters in a variety of *Eimeria* models. However, translation to field settings remains constrained by variable bioactive composition, limited standardization, inadequate pharmacokinetic data, and the scarcity of large-scale, multi-farm validation studies. This review provides a concise summary of current evidence and delineates critical knowledge gaps to guide the development, optimization, and deployment of next-generation anticoccidial strategies. Together, natural products and emerging biotechnologies provide a promising foundation for sustainable, high-welfare, antibiotic-independent coccidiosis control.

## Simple Summary

Globally, coccidiosis is one of the most economically significant disease affecting the poultry industry. Over the years, growing concerns over drug resistance, regulatory restrictions on antimicrobial use on livestock production, and the demand for residue-free products has driven the accelerated interest in natural or non-antibiotic strategies to control coccidiosis. This review summarizes the current evidence of approaches ranging from phytochemicals to botanicals, nutritional and mineral modulators, microbiome-directed biotics and metabolites, immunological effectors, nanodelivery systems and omics-guided platforms. Collectively, these strategies intervene at multiple biological nodes, from a direct parasitic effect at different developmental stages of the parasite to preserving mucosal architecture and modulating host immunity, reprogramming gut microbial ecology and improving metabolic resilience during infection. This review provides a concise integration of current evidence and articulates critical gaps to guide the development of next-generation, sustainable anticoccidial strategies.

## 1. Introduction

In 2024, global egg production reached 97.38 million tonnes and poultry meat production rose to 148.18 million tonnes, making poultry the leading contributor to the global meat supply [1]. More recent projections suggest that egg (in the shell, preserved or cooked) consumption will rise to 108.35 million tonnes by 2034, while poultry meat will account for roughly 62% of global meat consumption, corresponding to 173 million metric tonnes of ready-to-cook products. This reaffirms the central role of the poultry industry in supporting food security with an affordable, high-quality protein supply [1,2]. Over the years, the poultry sector has expanded significantly to meet the demand of the growing human population. However, the industry is constantly challenged by several disease outbreaks, rising feed and labor costs, regulatory and trade policies, and environmental issues that affect productivity, animal health, economic viability, and environmental sustainability [1,3]. Coccidiosis, an intestinal disease caused by an obligate intracellular apicomplexan parasite of the genus *Eimeria*, is the most economically devastating parasitic disease affecting the poultry industry worldwide, and causes microscopic and macroscopic intestinal pathologies, acute hemorrhagic enteritis, significant malabsorption, poor feed conversion, reduced growth, and mortality in infected chickens [4,5,6,7]. Recent estimates indicate a global economic burden of £10.36 billion for coccidiosis-induced production losses, including associated prophylaxis and treatment, based on 2016 prices, rising to approximately £12.9 billion by 2022 [8,9].

Since the first report on the effectiveness of sulfanilamide against coccidiosis in 1939, several synthetic anticoccidials and ionophores have been developed and are routinely used as chemoprophylactic agents, often in conjunction or in rotation with live or attenuated vaccines to mitigate drug resistance and sustain flock performance [10,11,12,13,14,15]. However, the long-term efficacy of nearly every anticoccidial compound introduced has progressively decreased over the years due to the emergence of drug-resistant *Eimeria* spp., undermining the performance margin of drug-based control programs [13,16,17,18,19,20]. Significantly, these strategies neglect the broader immunometabolic and ecological perturbations accompanying *Eimeria* infection. Conversely, although live vaccines are effective in inducing protective immunity, these vaccine platforms often face strain-specific protection, and they are limited by variable immunogenicity, high production costs and lengthy, labor-intensive manufacturing timelines [21]. At the same time, regulatory constraints on in-feed antimicrobials and growing consumer preference for antibiotic-free production systems have intensified the demand for residue-free and non-pharmacological disease control strategies [22,23,24].

From a pathobiological standpoint, *Eimeria*-induced enteric damage extends far beyond localized intestinal epithelial barrier injury and disruption of tight junction integrity to the induction of oxidative stress, immune dysregulation, and inflammatory pathology. These insults collectively impair nutrient absorption, compromise mucosal function, and predispose chickens to dysbiosis-mediated bacterial enteropathies such as necrotic enteritis (NE) in the field [6,25,26]. The resulting interplay between epithelial injury, microbial imbalance, and sustained inflammation amplifies performance losses and increases reliance on intensive anticoccidial programs alongside antibacterial interventions for secondary infections. This antimicrobial exposure intensifies selection pressure within the gut, thereby accelerating the emergence and dissemination of antimicrobial resistance (AMR). Consequently, mitigating epithelial damage during *Eimeria* infection and restoring gut homeostasis are now recognized as critical determinants of disease resilience, performance stability, and antibiotic stewardship in modern poultry systems.

The combined challenges of drug resistance, regulatory restrictions, and dysbiosis-associated sequelae delineate a sustainability issue that cannot be resolved by single-target chemotherapeutics alone, as drug-based approaches primarily target parasite replication but are constrained by rapid development of resistance, the multi-species nature of coccidiosis, and their inability to fully restore epithelial integrity, normalize gut microbial balance, or resolve host immune dysfunction underlying disease recurrence and productivity losses [5,11,12,22,25,26]. This underscores the urgent need to develop more integrated, diverse, and ecologically sustainable alternatives that extend beyond traditional drug-based interventions that can suppress parasite development and mitigate downstream immunopathological consequences on gut health and performance. Building on this premise, this review describes the expanding landscape of non-antibiotic and emerging strategies for sustainable coccidiosis control that transcends approaches beyond ionophores, chemical anticoccidial drugs and *Eimeria* vaccines.

**Figure 1:**
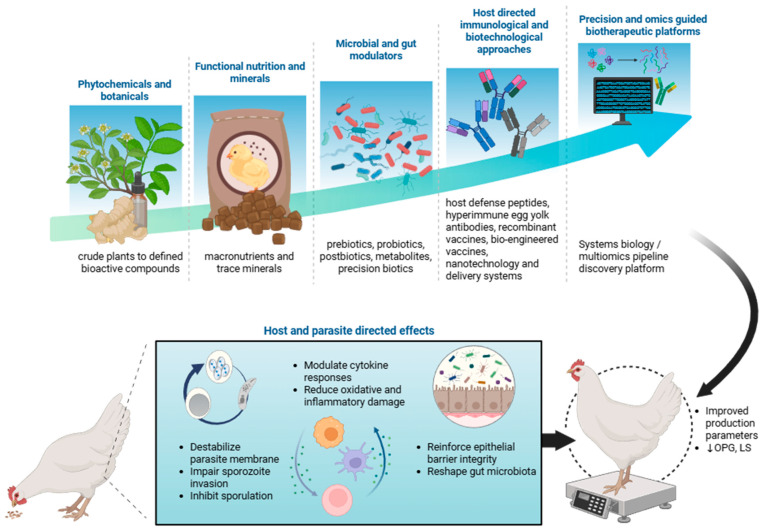
Conceptual framework of non-antibiotic and emerging strategies for *Eimeria* infection control in poultry. The schematic illustrates a continuum of complementary control approaches in coccidiosis control strategies highlighting the parasite-directed effects and host-mediated protective effects of the strategies. Image is created in Biorender with publication license. ↓ indicates reduction; OPG, oocyst per gram; LS, lesion score.

Herein, we provide a conceptual framework by classifying the interventions into five interlinked domains that reflect both mechanistic and technological progression: (i) phytochemicals and botanicals, ranging from crude plant extracts to defined bioactive compounds; (ii) functional nutrition and mineral modulators that optimize host metabolism, epithelial repair, and oxidative balance; (iii) microbial and gut modulators, including biotic components (e.g., prebiotics, probiotics, postbiotics, and synbiotics), metabolites and precision biotics that support intestinal integrity and maintain intestinal homeostasis; and innovative technologies including (iv) host-directed immunological and biotechnological approaches that harness or augment innate and adaptive immune defenses and (v) precision and omics-guided biotherapeutic platforms that integrate system biology, bioinformatics and molecular design to increase potency, targeting and reproducibility (Figure 1). Google scholar and PubMed search engines were used to search for articles containing the keywords “coccidiosis,” “broiler,” “poultry,” and “*Eimeria*” in combination with “strategies,” “control,” or “non-antibiotic”. Representative examples of these approaches and their demonstrated effects are summarized in the tables. Here we provide concise, evidence-focused profiles and summaries of these representative agents and their documented effects on oocyst shedding (OPG), intestinal lesions (LS), and performance parameters body weight gain (BWG) and feed conversion ratio (FCR) in chicken coccidiosis models together with relevant translational and mechanistic outcomes, including carcass and meat quality traits, intestinal integrity, inflammatory responses, gut microbiota modulation and host physiological responses, where available. Beyond literature synthesis, this review offers strategic insight into the translational potential of non-antibiotic strategies, identifying promising approaches, rational combinations and key bottlenecks that constrain field implementation.

## 2. Phytochemicals and Botanicals

Phytochemicals, also called phytobiotics or phytogenics, and botanicals represent a vast and promising category of naturally plant-derived bioactive compounds, preparations or extracts being explored as alternatives to conventional antibiotics for their health-promoting and disease-mitigating properties with a comparatively low residue, no risk of resistance selection and minimal side effects in animal production [27,28]. From a mode-of-action perspective, these diverse plant-derived compounds display multifaceted, often complementary biological mechanisms. Many compounds exert direct anticoccidial effects by destabilizing parasite membranes, impairing sporozoite invasion and intracellular development or inhibiting the sporulation of oocysts, while others confer host benefits by modulating cytokine responses, dampening oxidative and inflammatory damage, reinforcing epithelial barrier integrity, and reshaping the gut microbiota to a more protective profile [27,29]. Throughout this section, in vitro observations including oocyst wall disruption, sporulation inhibition, sporozoite viability reduction, and invasion assays are presented as supportive mechanistic indicators of antiparasitic potential, whereas anticoccidial efficacy is primarily interpreted based on biologically and translationally relevant in vivo outcomes such as OPG, LS, growth performance and recovery parameters. These activities arise from several major classes of active ingredients, including essential oils (e.g., carvacrol, thymol, eugenol, cinnamaldehyde), targeted herbal extracts (e.g., allicin, artemisinin derivatives, curcumin, berberine); and a broader class of phenolics (e.g., flavonoids and tannins), terpenoid glycosides (e.g., saponins), and macromolecular bioactives (e.g., lectins and polysaccharides) (Table 1).

### 2.1. Essential Oils

Essential oils (EO) are volatile, aromatic secondary metabolites extracted from various plant species with diverse bioactive properties and well-recognized antibacterial, antioxidant, and immunomodulation properties [30]. In poultry, EOs have received increasing attention as phytogenic feed additives to improve growth performance and mitigate infections. Among EOs, the phenolic monoterpenes carvacrol and thymol derived from oregano (*Origanum vulgare*) and thyme (*Thymus vulgaris*) have emerged as potent natural anticoccidials with a multifaceted mode of action involving both direct parasiticidal activity and host-mediated protective effects [31,32,33,34,35]. In vitro studies provide mechanistic evidence that thyme and oregano EOs exert direct effects on *Eimeria* developmental stages. Specifically, under in vitro conditions, thyme EO disrupted the oocyst wall of a mix suspension of *Eimeria* spp. oocysts with a reported median lethal concentration (LC_50_) of 0.315 mg/mL, indicating a direct oocysticidal effect primarily relevant to experimental or environmental context [31]. More importantly, both oregano and thyme (40 ppm) EOs, and to a lesser extent their main active compounds, thymol and carvacrol (20 ppm), inhibited *Eimeria tenella* (*E. tenella*, ET) sporozoite invasion and interfered with the intracellular development of schizonts in Madin-Darby bovine kidney (MDBK) cells [32]. In vivo, dietary supplementation with oregano EO significantly reduced OPG and LS in *Eimeria* spp.-infected broilers, leading to improved BWG and FCR [33,34]. Beyond effects on growth parameters, oregano EO supplementation improved carcass traits and ameliorated parasite-induced intestinal damage by reinforcing epithelial integrity with the upregulation of tight junctions such as zonula occludens-1 (ZO-1) in the duodenum and claudin-1 (CLDN-1) expression in the duodenum and ileum of *Eimeria*-challenged broilers [35]. However, an earlier report found that despite improvements in villus height, crypt depth, digestive enzyme activity and antioxidant status with dietary oregano EO supplementation during mix *Eimeria* infection, it did not achieve BWG or FCR outcomes comparable to monensin and showed no synergistic or additive effects when used in combination with an ionophore anticoccidial [36].

Eugenol, a dominant phenolic in clove (*Syzygium aromaticum*) EO also has antioxidant, antimicrobial, anti-inflammatory and antinociceptive properties [37,38]. In healthy broilers, clove EO treatment improved duodenum ZO-1 expression and breast yield, whereas, in a mixed *Eimeria* spp. challenge model, dietary supplementation with clove EO at 500 ppm improved recovery drip loss in the breast muscle of broilers and resulted in significant overall BWG compared to control diet over a 42-day trial following challenge on day 10 [35]. Additionally, eugenol significantly reduced OPG and LS in *E. tenella* (ET)-infected broilers, supporting biologically relevant in vivo activity [39]. Complementary in vitro studies showed that clove EO disrupted oocyst wall of mix *Eimeria* spp., with a reported LC_50_ of 0.543 ± 0.304 mg/mL [31]. More recently, betelvine (*Piper betle*) leaf essential oil containing eugenol has shown a complementary parasite-directed mechanism by inhibiting sporulation and disrupting ET oocysts in vitro [29]. Interestingly, eugenol showed inferior and limited activity in vitro in reducing the sporulation rate and structural disruptions of mixed *Eimeria* spp. oocysts compared to thymol and carvacrol [40].

Another widely studied EO constituent, cinnamaldehyde (CINN) from cinnamon (*Cinnamomum* spp.), also exerts robust antioxidant and antimicrobial effects while preserving intestinal histomorphology and barrier integrity [41]. Broilers fed with CINN increased expression of IL-1β, IL-6, IL-15 and IFN-γ in the intestine and improved BW in *E. maxima* (EM)-infected broilers and improved BWG and reduced LS and OPG in *E. acervulina* (EA)-infected broilers [42,43]. In vitro, CINN induced cell proliferation in chicken spleen lymphocytes, increased levels of nitric oxide in macrophages, inhibited the growth of tumor cells and reduced the viability of ET sporozoites [42]. More specifically, CINN reduced OPG, increased antibody response to EtMIC2, resulting in a moderate anticoccidial index (ACI), and improved FCR and meat quality during ET challenge in broilers [42,44,45]. Collectively, EOs represent a promising phytogenic anticoccidial that acts with combined parasite-directed and host-protective mechanisms. However, the inherent variability in composition and stability limits consistency in field conditions.

**Table 1:** List of phytochemicals and botanicals control strategies for coccidiosis with their anticoccidial effects in broilers.

Control Strategy/ Approach	Representative Sources/Products	Main Bioactive Components	Effects on Coccidiosis	References
Essential oils	**Oregano:** *Origanum vulgare*, **Thyme:** *Thymus vulgaris*	Carvacrol, Thymol	**ET:** inhibit sporozoite invasion and interfere schizont development in vitro; **Mix** ***Eimeria*** **spp.:** Oregano oil: improve FCR, improve breast and tender yield, enhanced ZO-1 expression in duodenum, enhanced CLDN-1 expression in duodenum and ileum, reduces OPG, improved BWG, reduced LS; Thyme: lysis of *Eimeria* spp. oocysts	[31,32,33,34,35]
	**Clove:** *Syzygium* *aromaticum*	Eugenol	**ET:** reduced OPG and LS, inhibit sporulation, lysis of oocysts in vitro; **Mix** ***Eimeria*** **spp.:** lysis of oocysts, improve BWG, improve breast and tender yield	[29,31,35,37,38,39]
	**Cinnamon:** *Cinnamomum* spp. (*Cinnamomum verum*, *C. cassia*)	Cinnamaldehyde	**EA:** increased BW, reduced OPG, reduced LS; **EM**: increased BW; **ET**: reduced sporozoite viability, increased antibody response to EtMIC2; reduce OPG, reduce LS, increase BWG, improve FCR and meat quality	[42,43,44]
Herbal extracts and plant-derived bioactive compounds	**Garlic:** *Allium* *sativum*	Allicin	**ET:** reduce LS and OPG, increase BW, improve ACI, promote immunoglobulin secretion, oocyst deformation, inhibit sporulation, inhibit sporozoites	[46,47,48]
	**Wormwood:** *Artemisia* spp. (*Artemisia annua*, *A. afra*, *A. seiberi*)	Artemisinin derivates	**EA:** reduced OPG; **EM:** not significant OPG and LS reduction; **ET:** impair oocysts wall formation and sporulation, reduced LS, reduced OPG, reduced mortality, improved BWG and FCR; **Mix** ***Eimeria*** **spp.:** reduced OPG, improved FCR, modulate positive gut microbiota, oocyst sporulation inhibition	[49,50,51,52,53,54,55,56,57,58,59]
	**Chinese goldthread:** *Coptis chinensis*; **Asian barberry:** *Berberis asiatica*; **Indian barberry:** *Berberis lycium*	Berberine (Isoquinoline alkaloid)	**EA:** reduce BW, OPG and LS, increase villus height in the duodenum; **E. mi:** reduce OPG; **EM:** reduce OPG at 0.5% supplementation but reduce BW; **EP:** reduced OPG; **ET:** reduced OPG, improve BW, attenuate oxidative stress	[60,61,62,63,64,65]
	**Turmeric:** *Curcuma longa*	Curcumin (polyphenolic diferuloymethane)	**EA:** not significant reduction in OPG, increase BW, not significant EtMIC2 serum antibody when supplemented alone; **EM:** increase BW, reduced LS, increased anti-EtMIC2 serum antibody, increase spleen cell proliferation, reduced OPG, reduce plasma NO; **ET:** increase BW, improve FCR, reduced OPG, increase spleen cell proliferation, induce morphological changes and reduce sporozoite viability in vitro and infectivity on MDBK cells, reduce LS; **Mix** ***Eimeria*** **spp.:** increase villus height, crypt depth and villus:crypt ratio, reduced leucocytes and lymphocytes, reduce ROS and reactive to TBARS, increase SOD, increase total PUFA, reduced total SFA, reduce OPG	[49,65,66,67,68,69,70,71,72,73]
	**Neem:** *Azadirachta indica*	Azadirachtin, salanin, numbin, nimbidin, meliantrial, polyphenols (flavonoids: quercetin, kaempferol, rutin, catechin; Phenolic acids: gallic, chlorogenic, caffeic, coumaric, ellagic, rosmarinic), tannins, saponins, triterpenes/sterols	**ET:** inhibit sporulation in vitro, reduced OPG, improve BW; **Mix** ***Eimeria*** **spp.:** reduced mortality and OPG, improved FCR	[74,75,76,77,78]
	**Cobbler’s pegs/Beggar’s tick/Black Jack/Spanish Needles:** *Bidens pilosa*	Polyphenols/flavonoids (mixture)	**ET:** improved BWG and FCR, reduced gut pathology, elevated beneficial gut bacteria, reduced OPG, reduced mortality rate, inhibited oocyst sporulation, sporozoite invasion and schizonts, enhanced T-cell mediated immunity, upregulated expression of ZO-1 and IL-6, increased antioxidant enzymes and peptide transporter 1; **Mix** ***Eimeria*** **spp.:** Improved BWG, FCR, lower mortality, reduced OPG and gut pathology, improved ACI	[79,80,81,82,83]
	**Mojave yucca/Spanish Dagger:** *Yucca schidigera*; **Soap bark:** *Quillaja saponaria*;	Saponins	**EA:** reduced LS, improved BW; **EM:** improved BW; **ET:** improved BW, reduced LS; **Mix** ***Eimeria*** **spp.:** downregulate expression of IL-1β, reduced OPG, reduced LS, improve BW and FCR, increase ileal morphometrics	[84,85,86,87,88]
	**Grape seed:** *Vitis vinifera*, **Pine Bark:** *Pinus radiata*	Condensed Tannins (Proanthocyanidins)	**ET:** increase BWG, decrease mortality, reduce LS, reduce plasma NO, MDA and decrease SOD	[89]
	**Gall nuts:** *Rhus chinensis*; **False daisy/Kameng:** *Eclipta prostrata*	Hydrolysable Tannins (Tannic Acid, Gallic acid)	**Mix** ***Eimeria*** **spp.:** reduced OPG and LS, inhibit sporulation	[90,91]
	Myricetin, Quercetin, Apigenin, Xanthohumol	Flavonoids	**Mix** ***Eimeria*** **spp.:** reduce LS and OPG, reduce oxidative and lipid peroxidation markers (i.e., hydrogen peroxide, H_2_O_2_; ROS; MDA), increase total antioxidant capacity, reduce expression of inflammatory biomarkers (i.e., IL-1β, IL-6, TNF-α, CCL20, CXCL13 and avian defensins AvBD16), disrupt oocysts and reduce oocyst number	[92,93]
	**Shiitake:** *Lentinus edodes*, **White jelly:** *Tremella fuciformis*, **Huang Qi:** *Astragalus membranaceus radix*	Polysaccharides	**ET:** reduced OPG, improved BW, more positive effect when in conjunction with coccidia vaccine	[94]
	**Wood-rotting mushroom:** *Fomitella farxinea*	Lectins	**EA:** improve BW, reduced OPG	[95]
Synergistic Blends	Oregano + Thyme + Garlic EO	Carvacrol, thymol, Sulfuric Compounds	**Mix** ***Eimeria*** **spp.:** improve BW, growth rate and FCR; reduced OPG	[96]
	Oregano + *Citrus* spp. EO	Polyphenols and terpenes/terpenoids	**Mix** ***Eimeria*** **spp.:** low BWG, poor FCR, reduced OPG, increased BCFA in the ceca	[97]
	Eucalyptus oil + Apigenin + Eugenol EO	Flavonoids and Essential oils	**ET:** reduced OPG and LS, improve BWG, improve ACI and FCR	[39]
	Green tea extract, cinnamon oil, pomegranate extract	Catechin, Cinnamaldehyde, Polyphenols	**EM:** enhanced BW gain, reduced oocysts shedding, decreased pro-inflammatory cytokines (IL-1β, TNFSF-15 and IFN-γ)	[98]
	*Azadirachta indica*, *Nicotiana tabacum*, *Calotropis procera*, *Tachyspermum ammi*	Alkaloids, saponins, flavonoid	**ET:** improved BWG, reduce OPG and LS, improve survivability	[99]
Commercial blends	Apacox^®^	*Agrimonia eupatoria*, *Echinacea angustifolia*, *Ribes nigrum*, *Cinchona succirubra*	**ET:** improve BWG and FCR, reduced OPG	[100]

ACI, anticoccidial index; BCFA, branched-chain fatty acid; BW, body weight; BWG, body weight gain; CLDN-1, claudin-1; FCR, feed conversion ratio; EA, *Eimeria acervulina*; EM, *E. maxima*; E. mi, *E. mitis*; EO, essential oil; IL, interleukin; LS, lesion score; MDA, malondialdehyde; MDBK, Madin-Darby bovine kidney cells; NO, nitric oxide; OPG, oocyst per gram; ROS, reactive oxygen species; TBARS, thiobarbituric acid; SOD, superoxide dismutase; PUFA, polyunsaturated fatty acids; SFA, saturated fatty acids; ZO-1, zonula occludens 1.

### 2.2. Herbal Extracts and Plant-Derived Bioactive Compounds

In addition to volatile essential oils, a wide array of volatile and non-volatile phytochemicals derived from various herbal extracts exert potential anticoccidial effects through distinct and overlapping mechanisms.

#### 2.2.1. Garlic (*Allium sativum*)

One well-characterized compound is allicin, the principal organosulfur component of garlic [101,102]. Allicin has a broad-spectrum antimicrobial property along with significant anti-oxidant, immunomodulatory, intestinal gut microbiota modulation, and anti-inflammatory effects [102,103]. In coccidiosis, dietary allicin has demonstrated efficacy against ET by reducing intestinal LS and OPG, improving BWG and ACI, and promoting host immunoglobulin secretion, highlighting both parasite suppression and immune improvement [46,47]. In vitro, allicin inhibited sporozoite infection in MDBK cells, caused oocyst deformation and leakage of oocysts contents, and reduced the number of oocysts and sporulation of ET [47,48]. However, dietary supplementation with a high dose of allicin (0.50 mL/kg) has toxic effects on the liver of layer chicks [104].

#### 2.2.2. Wormwood (*Artemisia* spp.: *Artemisia annua*, *A. afra*, *A. seiberi*)

Extracts from *Artemisia* spp. have received substantial attention, primarily due to the presence of artemisinin and its derivatives, which are renowned for their antimalarial properties [49,105]. The direct parasitic interference of artemisia extracts occurs through inhibition of sarco/endoplasmic reticulum calcium ATPase (SERCA). It disrupts calcium homeostasis in the parasite, and with the induction of oxidative stress, impairs oocyst wall formation and subsequent sporulation of oocysts [50]. Likewise, the host-directed effects of this extract are related to promoting apoptosis of infected cells, suppressing NF-κB and IL-17A-mediated inflammation, providing oxidant protection, potentially supporting gut microflora, and improving intestinal barrier function with the upregulation of tight junctions such as occludin (OCLN) in the jejunum and ileum and ZO-1 in the jejunum of supplemented broilers [51,52]. In *Eimeria* spp. challenge models including mixed and single infection with EA and ET, artemisia was reported to reduce OPG, improve BWG and FCR, reduce LS and mortality, and positively modulate the gut microbiota, in addition to inhibiting sporulation and invasion in vitro [49,50,53,54,55,56,57,58,59,105,106]. Interestingly, *A. sieberi* and *A. annua* failed to show protective effects against EM [49,105].

#### 2.2.3. Berberine (Chinese Goldthread (*Coptis chinensis*) and Barberries (*Berberis asiatica*, *B. lycium*))

Berberine is an isoquinoline alkaloid with complementary pathogen- and host-directed mechanisms. In addition to having direct antimicrobial and antiprotozoal properties, berberine modulates immunity by attenuating NF-κB-dependent inflammatory signaling, reducing excessive pro-inflammatory cytokine production, and restoring redox balance via enhanced antioxidant defenses [60,61,62,107]. Concurrently, berberine supports intestinal integrity by increasing villus height and villus height and crypt ratio in addition to reshaping gut microbial composition towards protective taxa [60,61,62,107]. In broilers outside *Eimeria* challenge, dietary berberine consistently reduced acute inflammatory responses, improved systemic and mucosal antioxidant capacity and improved indices of intestinal integrity and microbial composition in context-dependent gains in growth performance under production stress [61,62,107]. Earlier work with yellow-feathered broilers showed that berberine improved growth performance and remodeled cecal microbiota/functions, consistent with microbiota-mediated effects. Specifically, BBR treatment decreased the relative abundance of Firmicutes-associated taxa (i.e., *Lachnospiraceae, Lachnoclostridium, Clostridiales* and *Intestinimonas*) while increasing *Bacteroidetes* genus *Bacteroides*. Functional prediction revealed enriched pathways related to metabolism, organismal systems and genetic information processing. Notably, broiler growth performance positively correlated with the abundance of phylum *Bacteroidetes*, and beneficial genera such as *Bacteroides* and *Lactobacillus* in the cecal contents [108]. A more recent study found that high doses of berberine in broilers improved gut-wall morphology despite the expansion of *Enterobacteriaceae* and a reduction in beneficial taxa, suggesting host/epithelial pathway effects that are not strictly microbiota-composition-dependent in this study [109].

In *Eimeria* infection model, berberine shows consistent efficacy across species with design-specific readouts. In single-species EA infection, berberine lowered OPG and LS, increased duodenal villus height, and modulated cell-integrity and immune- and cellular homeostasis-related genes [60]. More broadly, OPG reductions were also reported for *E. mitis* (Emi), *E. praecox* (EP), and ET without affecting body weight with 0.2% berberine supplementation. In EM infection, berberine supplementation of 0.2% proved ineffective, while a 0.5% dose significantly reduced OPG at the expense of compromised BWG [63]. In ET infection, berberine normalizes copper and zinc homeostasis and attenuates oxidative stress by decreasing lipid peroxidation and increasing superoxide dismutase (SOD) activities [110,111]. Based on fractions, the methanolic root bark extract of *Berberis lyceum* outperformed hexane and aqueous fractions in improving BW and reducing OPG in ET-infected broilers [112]. Berberine also has synergistic anticoccidial activity with amprolium at a 1:1 ratio [64].

#### 2.2.4. Curcumin (Turmeric: *Curcuma longa*)

Curcumin, the principal polyphenolic diferuloylmethane from turmeric (*Curcuma longa*), exhibits anticoccidial activity with parasite-directed action, including reducing sporozoite viability, and significant host modulation [65]. It exerts potent anti-inflammatory effects partly by blocking the NF-κB activation pathway and suppressing inflammatory gene expressions (e.g., TNF-α, IL-1β, IL-6, IL-8, CD28, CD200, FGF2, FGG, IL10-RB), while concurrently enhancing host immune competence by stimulating spleen lymphocyte proliferation and upregulating in macrophages the expression of IL-12 and IL-18, key cytokines that support Th1-biased, cell-mediated immunity critical for intracellular parasite control [66,67,113]. Moreover, curcumin mitigates oxidative stress and protects cells from lipid peroxidation. In coccidia challenge, dietary supplementation of curcumin resulted in improved BWG and FCR, reduced OPG and LS, and increased spleen cell proliferation in EM- and ET-infected chickens, in addition to increasing anti-EtMIC2 antibody response and lowering plasma nitric oxide (NO) [49,66,68,69]. Histologically, turmeric supplementation (300 mg/kg body weight) nearly eliminated cecal intestinal pathologies such as submucosal crypt and gland degeneration and necrosis caused by intracellular ET developmental stages, with only rare residual degenerated schizonts observed two weeks post-infection [69]. In EA-infected supplemented broilers, increased BW was observed, but there were no significant effects for LS, OPG and EtMIC2 antibody [70,71]. In mix *Eimeria* spp. infection, curcumin (100 mg/kg) supplementation in broilers reduced OPG and improved intestinal morphology as reflected by increased villus height and crypt depth ratio. While serum antioxidant enzyme activities were largely unchanged, curcumin significantly attenuated oxidative stress in breast muscle by reducing reactive oxygen species (ROS) and lipid peroxidation (thiobarbituric acid-reactive substances, TBARS) while increasing SOD activity, and favorably modulating meat fatty acid composition toward higher total polyunsaturated fatty acids (PUFA) and lower total saturated fatty acid [72]. Complementary evidence from naturally *Eimeria* spp. infected laying hens demonstrated significant reduction in OPG at curcumin inclusion levels of 30- and 50 mg/kg, together with improved oxidative stability of eggs reflected by lower TBARS and increased total antioxidant capacity (TAC) in egg yolk during storage [73].

#### 2.2.5. Neem (*Azadirachta indica*)

This plant has long been recognized as having potent insecticidal activity with little or no mammalian toxicity, and recent studies have expanded its relevance as an antimicrobial, anti-inflammatory and anticoccidial agent [74,75]. Its hallmark limonoid azadirachtin is the primary bioactive component, complemented by salamin, nimbin, nimbidin, meliantrial, and a variety of flavonoids (e.g., quercetin, kaempferol, rutin, catechin), phenolic acids (e.g., gallic, chlorogenic, caffeic, coumaric, ellagic, rosmarinic), tannins, saponins, and triterpenes/sterols, each with bioactivity related to direct parasite interference or host-directed mucosal support [75,76]. Neem demonstrates functional anticoccidial efficacy across models. In an ET infection model, neem supplementation inhibited sporulation in vitro and reduced OPG and improved BWG in vivo [76,78]. Similarly, in mixed *Eimeria* spp. infections, dietary supplementation improved the feed conversion ratio (FCR) and reduced OPG and mortality in broilers [114].

#### 2.2.6. Cobbler’s Pegs (*Bidens pilosa*, BP)

BP extracts, rich in polyphenols and flavonoids, have distinct anticoccidial mechanisms primarily focused on disrupting multiple stages of the parasite lifecycle, from the inhibition of sporulation to sporozoite invasion and schizont maturation, without necessarily causing direct killing [79]. This effect is complemented by significant host benefits that include improved T-cell-mediated immunity, improved intestinal barrier integrity by upregulation of ZO-1, restoration of morphology by improved villus/crypt ratio, and modulation of gut microbiota toward beneficial probiotic taxa while decreasing pathogens [79,80,81,82]. For ET and mixed *Eimeria* spp., BP supplementation improved BWG and FCR, reduced mortality, OPG and gut pathology, and improved antioxidant enzyme activities [80,82,83].

#### 2.2.7. Saponins

These are a broader class of phytochemicals with amphiphilic glycosides naturally occurring in various plants and acting as emulsifiers with immunomodulatory, anti-inflammatory, antimicrobial, antioxidant, antiviral and hypocholesterolemic properties that have pharmaceutical, cosmetic and food industry applications [115]. Common saponin sources include Spanish dagger (*Yucca schidigera*, Y) for steroidal saponins associated with anti-inflammatory and antioxidant activity that are widely used in feed/food, and soap bark (*Quillaja saponaria*, Q) for triterpenoid saponin used as natural emulsifiers and immunostimulatory vaccine adjuvants [115,116]. In broilers, supplementation of yucca at 125 mg/kg in feed significantly improved body weight, production parameters, and carcass quality [84]. Comparative analysis with pure yucca saponin extract and a whole plant Y and Q-supplemented broiler diet showed increased serum immunoglobin (IgA, IgY, IgM) and total antioxidant capacity levels in addition to improved VFAs (acetic acid, butyric, and valeric) in cecal contents, and reduced contents of ammonia nitrogen, IL-6, TNF-α, and malondialdehyde (MDA) in the serum of supplemented broilers [117]. In a coccidiosis model, yucca-derived saponin at 250 mg/kg supplementation in mixed *Eimeria* spp. infected broilers showed a similar lymphocyte count at 7-DPI, jejunum mucosal thickness, and downregulated IL-1β mRNA expression in the duodenum and cecal tonsils similar to uninfected broilers [85]. Interestingly, in battery trials with EA-infected broilers, the combined effect of Q+Y improved BWG and reduced LS compared to infected group, but there was no significant difference for OPG. In single species infection, supplementation improved BWG in EM-infected broilers and improved BWG and reduced LS for ET-infected broilers [86]. In a subsequent floor pen trial with mixed *Eimeria* spp., supplementation of Q+Y at 250 ppm significantly improved FCR and BWG at day 42, reduced OPG at day 21 and day 28, and reduced LS at day 28. These effects were further improved when Q+Y were administered with salinomycin (66 ppm) [86]. Vaccinated birds fed with Q+Y reduced coccidial exposure but maintained coccidial protection, improved BWG and FCR, and reduced mortality compared to their vaccinated controls [86,87]. Supplementation of Q+Y in broilers reared in litter with *Eimeria* oocysts have resulted in increased ileal villus height and decreased crypt depth, resulting in an increased absorptive surface [88]. A more recent study showed that the protective effects of the combination of Q+Y at 0.11 g/kg supported intestinal integrity and modulated mucosal immune response beyond the peak of coccidiosis infection to recovery phases of infection in broilers [118].

#### 2.2.8. Tannins

These are a major class of water-soluble polyphenolic secondary metabolites found widely in plants and are broadly categorized into hydrolysable tannins such as tannic acid from gall nuts (*Rhus chinensis*) or gallic acid from false daisy (*Eclipta prostrata*), and condensed tannins such as proanthocyanidins from grape seed (*Vitis vinifera*) or pine bark (*Pinus radiata*) [119,120,121]. Historically viewed as anti-nutrients due to protein binding, tannins are now recognized for having significant biological activities including antimicrobial, antioxidant, anti-inflammatory, and immunomodulatory effects relevant to gut health [122]. Their anticoccidial potential stems from multiple mechanisms, including protein binding interference with parasite enzymes or surface proteins that potentially hinders invasion or development [119]. An in vitro experiment confirmed that tannin can directly inhibit the sporulation of *Eimeria* spp. oocysts [90]. In mixed *Eimeria* spp. infection, tannic acid and tannic acid extract reduced OPG and LS [91]. Furthermore, their potent antioxidant and anti-inflammatory actions helped mitigate the severe oxidative stress and intestinal tissue damage induced by coccidia infection by reducing plasma NO, MDA and SOD levels and contributing to reduced LS and faster recovery of the gut mucosa in ET-infected proanthocyanidin extract supplemented broilers [89]. However, mixed results were described for the efficacy of tannic acid in coccidia vaccinated infected broilers [91,123]. Tannic acid can positively influence the intestinal microbiota composition, villus morphology and barrier function of the intestine, but the concentration dose must be kept below 0.375% or between 500–900 mg/kg in broilers [124,125].

#### 2.2.9. Flavonoids

These constitute a large and diverse group of polyphenolic secondary metabolites ubiquitous in fruits, vegetables and herbs, with prominent examples including quercetin and myricetin [126]. Their primary roles relevant to poultry lie in their potent antioxidant, immunomodulation, anti-inflammatory, and gut microbiota modulation properties that consequently improve growth-related parameters [127,128,129]. During *Eimeria* infection, significant oxidative stress and inflammation occur in the intestinal tissues. Flavonoids (e.g., myricetin) effectively reduce oxidative and lipid peroxidation markers (e.g., hydrogen peroxide, H_2_O_2_; ROS; MDA) and increase total antioxidant capacity and reduce the expression of inflammatory biomarkers such as IL-1β, IL-6, TNF-α, CCL20, CXCL13, and avian defensins AvBD16, thereby protecting broilers from coccidia-induced damage to subsequently improve growth parameters and reduce LS severity and OPG [92]. Quercetin was also found to exert a direct effect on mixed *Eimeria* spp. oocysts [93].

#### 2.2.10. Complex Polysaccharides

Complex polysaccharides, particularly β-glucans and heteropolysaccharides derived from mushrooms (e.g., shiitake: *Lentinus edodes*, King Oyster mushroom: *Pleurotus eryngii*, white jelly: *Tremella fuciformis*) and certain herbs (e.g., Huang Qi: *Astragalus membranaceus*) also function as immunomodulators and gut modifiers in poultry [67,130,131,132]. Unlike compounds with direct parasiticidal action, these polysaccharides interact with host immune cells to trigger signaling cascades that improve the innate immune response. In a previous report, treatment with shiitake extract or king oyster mushroom glucan resulted in the increased proliferation of chicken spleen lymphocytes, whereas in HD11 cells it increased the production of NO and cytokines (e.g., IL-1β, IL-4, IL-6, IL-8, IL-10, IL-12, IL-18, and TNFSF15) and phagocytic activity [20,133]. In ET infection, dietary mushroom and herb polysaccharides supplementation used in isolation in challenged broilers resulted in reduced BWG and increased OPG and LS compared to the vaccinated-challenged group. However, these polysaccharides, when used in conjunction with a coccidia vaccine, improved growth performance, and reduced the LS and OPG of challenged birds [94].

#### 2.2.11. Lectins

These are a structurally diverse group of carbohydrate-binding proteins found in various plants (seeds, roots) and fungi (e.g., wood-rotting mushrooms such as *Fomitella fraxinea*, FF) [134]. Their biological roles have been broadly described across biomedical contexts and include cellular signaling, cell-cell interaction in the immune system, host defense mechanism, inflammation, and metastasis [135]. In the context of enteric health and potentially coccidiosis, lectins have several modes of action, although their application as feed additives is complex due to potent antinutritional effects (e.g., binding to gut epithelium and interfering with nutrient absorption) [136]. However, some researchers found that FF-derived lectins are capable of stimulating lymphocyte proliferation and inducing NO secretion in HD11 cells, and the injection of FF-derived lectin in 18-day-old chick embryos with post-hatch EA challenge resulted in improved BW and reduced OPG, suggesting the anticoccidial potential of FF [95]. Further research regarding specific, nontoxic lectins or the low-dose application of lectins that could use these binding or immunomodulatory properties against *Eimeria* without causing gut damage is an area that requires further investigation for practical poultry application in the field.

Collectively, all the aforementioned phytochemicals target *Eimeria* at multiple stages while fortifying epithelial and immune defenses, but variability in source, batch-to-batch standardization, dose optimization, and matrix effects remains a major translational bottleneck. Priorities for future work include chemically characterized formulations, pharmaco-kinetic informed dosing, stability strategies, and synergy testing in field-scale trials.

### 2.3. Combined Synergistic Phytochemical Strategies

Combining essential oils (EOs) and other phytogenic extracts can produce synergistic anticoccidial effects that surpass those of individual phytochemical components. Notable approaches are (a) formulated blends of multiple EOs, (b) hybrid mixtures of EOs with other herbal extracts, and (c) mixtures of herbal complexes. EO combinations: Oregano oil (rich in carvacrol and thymol) paired with thyme oil (also high in thymol) and garlic oil (rich in organosulfur compounds such as allicin) exemplify this synergistic effect. When administered together in broiler diets, these EO blends have shown improved efficacy against *Eimeria* spp. infection [96]. Similarly, a blend of oregano and citrus oils has been tested in broilers, and although growth performance was not markedly improved, this EO mix significantly reduced OPG and LS, in addition to increasing cecal concentrations of beneficial branched-chain fatty acids, indicating a favorable gut microbiome shift [97].

In addition to pure EOs, researchers have explored formulations that combine EOs with other botanicals (e.g., saponin-containing herbs or alkaloid-bearing extracts). For instance, the coadministration of eucalyptus oil, apigenin and eugenol essential oil in experimental ET in broilers showed improved BWG and reduced LS and OPG, subsequently resulting in moderate ACI when given together, with a high margin of safety [39]. Likewise, in EM infection, a combination of green tea extract, cinnamon oil and pomegranate extract improved BWG in addition to reducing OPG and the expression of pro-inflammatory cytokines IL-1β, TNFSF-15 and IFN-γ [98]. In one trial, a blend of herbal extracts from neem (*Azadirachta indica*), tobacco (*Nicotiana tabacum*), giant milkweed (*Calotropis procera*) and carom seed (*Tachyspermum ammi*) also positively alleviated the negative effects of ET infection in broilers [99]. Polyherbal formulations such as Apacox have also been developed as commercial anticoccidial supplements with promising positive anticoccidial effects [100].

In summary, synergistic phytogenic blends apply the multi-modal actions of each individual component including antiparasitic (oocysticidal, sporozoite membrane lysis), antimicrobial (reshaping gut microbiota), and anti-inflammatory effects to protect chickens from coccidiosis. These combinations can improve production parameters in infected birds more effectively than single additives, and show the potential of carefully crafted phytochemicals and botanical mixtures as natural coccidiosis control agents while also underscoring the need for rigorous formulation control, safety assessment, and reproducibility testing prior to commercial deployment.

## 3. Functional Nutrition and Mineral Modulators

In poultry, the practical key feed ingredients to meet the nutrient requirements for optimal performance are feed grains, protein sources, lipids, minerals, and vitamins. While proper nutrition cannot fully replace vaccines or anticoccidial drugs, specific nutrients or feed ingredients can support a bird’s resilience and recovery during coccidiosis infection. Recent research has explored adjusting dietary proteins and amino acid profiles, altering fat sources, increasing or modifying dietary fiber, and supplementing specific micronutrients to bolster gut health and immunity in poultry. This section will highlight recent findings on nutritional interventions around macronutrients and trace minerals that mitigate coccidial challenge and support immune function while minimizing the catabolic cost to birds (Table 2).

### 3.1. Macronutrients

Macronutrients, including proteins, carbohydrates and lipids, serve as the primary substrates for energy and metabolic machinery in poultry [154]. During coccidia challenge, the use of these resources shifts from muscle growth to immunometabolic defense [155]. This shift underscores the need for a dietary strategy that prioritizes functional amino acids for mucosal repair and acute-phase protein synthesis, modulates carbohydrate profiles to prevent gut stasis and bacterial overgrowth, and uses specific fatty acids to inflict oxidative stress on the parasite.

Protein nutrition plays a dual contrasting role in coccidiosis. It is essential for growth and immune function, but during enteric challenge, excessive or undigestible protein in the gut may exacerbate this disease with increasing secondary bacterial infections by the excessive proliferation of potentially pathogenic bacterial species. A previous report has shown that feeding large amounts of animal-origin protein, especially fish meal, in the poultry diet improved *Clostridium perfringens* proliferation and toxin production, and consequently predisposed birds to necrotic enteritis (NE) [156]. More recent work by Sung and Adeola (2025) highlights that the quantity and digestibility of dietary protein play a pivotal role in modulating the host response to mixed *Eimeria* spp. [137]. In their work, increasing the proportions of indigestible protein in broiler diets exacerbated infection by lower goblet cell count in the intestine, decreased ileal nitrogen digestibility, increased ileal indigestible nitrogen concentration, downregulated tight junction (OCLN) gene expression, and impaired the performance of infected broilers [137]. Conversely, a low crude protein diet (LBP, 16% crude protein) had no adverse effect on mixed *Eimeria* spp. infected birds compared to infected controls, but when supplemented in combination with threonine, arginine or glutamine, the ileal digestibility of these amino acids was improved, with increased villus height in the duodenum with threonine, decreased intestinal permeability and gene expression of amino acid (AA) transporters with arginine, and decreased expression of tight junction protein CLDN-1 with threonine and glutamine supplementation. However, LBP in combination with methionine and a mix of all the aforementioned amino acids exacerbated infection severity [138]. While several studies concur on the overall positive effects of functional AA supplementation during coccidiosis infection, a recent report indicated that higher dietary AA is associated with an increased incidence of footpad lesions in coccidia-vaccinated broilers [139,140,141,142,157].

Carbohydrates represent the biggest constituent of poultry diets, and carbohydrate nutrition for coccidiosis management involves maintaining sufficient dietary energy with digestible carbohydrates and using certain fibers to promote gut health. Starch is generally very digestible in broilers, but coccidiosis can reduce its digestibility by 18.8% [158]. In normal broiler chickens, exogenous supplementation of α-amylase improved growth performance and starch and energy digestibility in supplemented broilers [159]. Insoluble fibers improved gut morphology, organ growth, and nutrient absorption, whereas soluble fibers often increase intestinal viscosity and negatively affect nutrient digestibility [160]. Dietary supplementation of insoluble fiber such as sunflower hulls ameliorates the negative effects of mixed *Eimeria* spp. infection by improving BWG and FCR, reducing OPG and LS, and increasing intestinal morphometric attributes in supplemented broilers [143]. The effects of dietary cellulose on growth performance, nitrogen utilization, cecal microflora and digesta retention time in normal birds are dose-dependent, with beneficial outcomes observed at inclusion levels up to 3.5%, while levels exceeding 3.5 negatively affect these parameters regardless of bird age [161].

Coccidiosis also reduced the ileal digestibility of fats by 96.2%, thereby increasing the availability of certain oils or fats that can supply readily absorbed energy and modulate the gut environment. This is one approach to alleviate energy shortfalls during coccidiosis infection [158]. A recent comparative study demonstrated that broilers fed with 5% fish oil (n-3 fatty acid (FA)-rich) have better resilience to mixed *Eimeria* spp. infection compared to 5% soybean oil. The fish oil group had improved gut barrier integrity and reduced LS [144]. Interestingly, an earlier report also showed similar positive effects on LS of n-3FA-containing diets from flaxseed and menhaden oil during ET infection but not in EM, indicating species-specific effects [145]. However, more recent studies suggest that increasing dietary lipid concentration, particularly during the starter period, may aggravate the severity of *Eimeria*-associated pathologies, underscoring the need for further research to understand the exact mechanism and role of FA profiles in modulating disease outcomes [162,163].

Macronutrient intervention offers a promising nutritional strategy to mitigate the impact of coccidiosis in broilers by supporting immunometabolic demands. However, future research should still focus on optimizing the nutrient type, digestibility profile, and functional interactions of these components to improve host tolerance without exacerbating disease severity and inducing unintended trade-offs of gut health and performance in birds.

### 3.2. Trace Minerals

These are established modulators of poultry gastrointestinal health, metabolic functions, and growth performance efficiency [164]. Over the years, interest in the functional roles of trace mineral supplementation has increased, and they are currently used in higher amounts in an attempt to improve the gastrointestinal health of poultry. Supporting this trend, a recent study using a multicomplex mineral-based diet in broilers experimentally infected with EA showed promising anticoccidial effects, including decreased fecal oocyst shedding and increased BWG [150].

Moreover, the bioavailability of minerals is influenced by several factors, including their chemical form, total mineral concentration, feed processing, and potential interactions with other dietary components [165,166,167]. The challenge of using inorganic minerals lies in their tendency to form insoluble complexes in the lower gastrointestinal tract due to interactions between mineral cations and other feed components at a higher luminal pH [167]. The most common example of an insoluble complex is mineral-phytate complex, which decreases mineral bioavailability. In addition, organic or chelated forms of trace minerals consist of metal ions complexed with organic ligands such as amino acids, peptides, or polysaccharides [164,167,168]. As a result, chelated trace minerals have received considerable attention for their improved bioavailability relative to their inorganic form, a trend supported by several studies [151,152,169].

These advantages appear particularly relevant in the context of coccidial challenge. For instance, Chen et al. (2022) demonstrated that broilers supplemented with a methionine hydroxy-analogue bis-chelate mineral blend containing Zn, Cu and Mn, and challenged with coccidiosis, showed decreased expressions of pro-inflammatory cytokine IL-17A [151]. Similarly, Biabani et al. (2024) reported that advanced chelate technology-based trace mineral supplementation in broilers challenged with a mixed infection of EA, EM, and ET attenuated inflammation [152]. Their findings showed the decreased expression of NF-kB, and in turn its associated proinflammatory cytokines, IL-1β, IL-6 and IFN-γ, along with an increased expression of the anti-inflammatory cytokine TGF-β. These studies reinforce the immunomodulatory potential of chelated trace mineral supplementation during coccidial infections. Among the trace minerals most extensively investigated in poultry are Zinc (Zn), Copper (Cu) and Manganese (Mn).

#### 3.2.1. Zinc (Zn)

Zn is critical for numerous metabolic and physiological processes, including growth, reproduction, meat quality, and immune responses. The recommended value of dietary Zn with optimal effects in terms of BWG and feed intake in broiler chicks from hatch to 21 d of age is 84 mg/kg [170]. Broilers that received supplementation of 1000 ppm Zn co-challenged with *E. brunetti* (EB) and *Clostridium perfringens* (CP) resulted in significantly greater LS compared with the EB or CP challenged-only group regardless of Zn supplementation [171]. In a similar setup but different study, Bortoluzzi et al. (2019) [146] evaluated ZnSO4 versus organic Zn supplementation in broilers co-challenged with EM infection and CP. Between the two sources, organic Zn resulted in higher BWG and FCR compared to ZnSO4. Notably, in the same study, supplementation with organic Zn showed immunomodulatory effects. In jejunal tissue, mRNA expression was downregulated for pro-inflammatory cytokine IL-8 and upregulated for anti-inflammatory cytokine IL-10 [146]. This agreed with previous studies where organic Zn suppressed the expression of pro-inflammatory mediator NF-kB p65 and pro-inflammatory cytokines TNF-α and IL-1β, and improved the expression of anti-inflammatory mediator A20, MUC2 and IgA [172,173]. At 80 and 100 ppm Zn supplementation, the mineral supplementation did not improve BWG in broilers challenged with mix *Eimeria* spp. but intestinal permeability, LS in ceca and OPG were significantly reduced. A species-specific effect was also observed in OPG. In Zn-supplemented birds, significant OPG reduction was observed for EA, but EM was significantly increased compared to the infected-only group [147]. Another interesting insight found by Turk & Stephens (1966, 1967) [165,166] demonstrated that malabsorption of an obvious specific nutrient is also dependent on *Eimeria* spp. infection, such that EA equally affects Zn and oleic acid absorption, while *E. necatrix* (EN) infection and Zn absorption were much more severely affected than oleic acid absorption. These findings underscore the interaction between Zn metabolism and the *Eimeria* spp. and highlight the need to also consider the type of infection when assessing mineral use [165,166].

#### 3.2.2. Copper (Cu)

This is an essential trace mineral that supports efficient growth performance and various metabolic functions while also contributing to the host’s defense against oxidative stress [147,148,174]. Several studies have documented improvements in BWG, ADG and FCR in broilers supplemented with Cu in their diets. For example, growth performance benefits were reported for 200 mg/kg of tribasic copper chloride (Cu(OH)_3_CI) supplementation in a broiler diet [174]. Similarly, a study by Forouzandeh et al. (2021) demonstrated a positive influence on weight gain by using either copper sulfate (CuSO_4_) or dicopper oxide (Cu_2_O)) given at different concentrations (15, 75, and 150 mg/kg) [175]). Both of these studies only focused on the bacterial population in the ileum affected by Cu. In terms of coccidiosis, the ability of Cu to reduce OPG with its immunomodulatory function can likely be attributed to its antibacterial and antiviral properties [153,172,173,176,177,178,179]. In broilers challenged with ET, the inclusion of 400 mg/kg Cu(OH)_3_CI resulted in reduced LS and oocyst index value, indicating a strong protective effect on the intestines [148]. Conversely, a more recent report on broilers infected with mix *Eimeria* spp. found that Cu at 100 and 150 ppm did not improve BWG, LS, intestinal permeability and OPG [147]. Collectively, these suggest that the protective effects of Cu may be species-specific and highly dose- and context dependent, limiting its reliability as a standalone anticoccidial strategy in complex field relevant infections.

#### 3.2.3. Manganese (Mn)

Mn is also used in feed additives to boost immunity. In an intestinal injury model, Mn supplementation aided in reducing the bacterial burden and promoted a pro-inflammatory response during *Salmonella* infection, as indicated by the increased expression of IFN-γ in the spleen and decreased expression of IFN-γ and IL-12 in cecal tonsils [180,181]. These studies suggest that supplementation not only improves intestinal barrier integrity but also affects the splenic inflammatory response. The effect of Mn with cobalt (Co) added in combination with other trace minerals showed improved growth in EA-challenged chicks [149]. Other studies used a combination of Zn and Mn and showed an improved immune response in 35-day-old broilers, and a chelated mineral combination of Zn, Cu and Mn during coccidial challenge showed decreased expressions of IL-17A [151,182].

#### 3.2.4. Combination of Minerals (Mn)

Multiple studies have documented positive growth and health responses in broilers that received mineral supplementation in combination rather than individually. One of the most widely studied combinations of trace minerals is Cu and Zn, which has been evaluated in both healthy and *Eimeria*-challenged broilers [153,175,183]. In broiler chicks challenged with mix *Eimeria* spp., supplementation of a combination of Cu_2_O and ZnO reduced OPG, improved microscopic features in the jejunum and lower LS [153]. However, because the trace mineral levels used in the study did not exceed 250 mg/kg, Cu and Zn supplementation had no measurable effect on growth performance, unlike findings from studies where BWG improved at inclusion rates of 250 mg/kg [153,183,184]. Expanding on earlier findings by Southern & Baker (1983), the effects of a combination of 50 mg/kg Zn and/or 250 mg/kg Cu were evaluated in EA-infected chicks [153,183]. While Cu supplementation did not show significant differences between control and infected chicks, Zn supplementation improved BWG for treated and challenged chicks. Interestingly, the same study demonstrated that Zn interfered with Cu absorption, but not the other way around. In another study, supplementation of 150 mg/kg Cu from Cu_2_O and 160 mg/kg Zn from potentiated ZnO yielded a decreased amount of OPG, which is consistent with findings that Cu and Zn supplementation reduced oocyst shedding in broilers infected with EM, ET and EA [153]. This synergistic effect can be attributed to the increased membrane permeability of *Eimeria* induced by copper ions, but still requires further investigation since the direct anticoccidial effect of Cu and Zn have not been reported in poultry before [177,178]. A drawback of using Zn and Cu as a feed additive is that bacteria may also develop resistance against these minerals due to their antimicrobial properties [185]. This is an important consideration when using high levels of trace minerals in feed additives.

Taken together, trace minerals support intestinal integrity, immune modulation, and growth performance under coccidial challenge, and chelated forms show superior bioavailability and anti-inflammatory effects. However, responses vary by mineral source, dose and *Eimeria* spp., and high inclusion rates may raise concerns about toxicity and resistance. Further work should focus on optimizing mineral combinations and elucidating their mechanisms of action in host–parasite interactions.

## 4. Microbiome and Adjunct Gut Modulators

The gastrointestinal tract of chickens hosts a diverse and dynamic microbial community that plays an essential role in nutrient digestion, immune system maturation and development, and defense against pathogens [186]. *Eimeria* infections disrupt intestinal microbial homeostasis, and these microbiota shifts in the gut in turn influence the outcome of diseases. Therefore, strategies aimed at modulating the gut microbiota and gut environment present a promising alternative and complementary approach for parasite control to promote a more balanced and functionally stable microbial community and gut environment that is less conducive to parasite proliferation and subsequent pathology (Table 3).

### 4.1. Prebiotics

Prebiotics, such as mannan-oligosaccharides (MOS), inulin or fructooligosaccharides (FOS) and β-glucans from yeast or fungal cell walls, are non-digestible dietary components that promote the growth and activity of gut microbes already residing in the host [205]. Broilers supplemented with MOS and β-glucans improved the relative abundance of *Lactobacillus* in ileal mucosa [206]. FOS supplementation in broilers resulted in increased and more diverse microbiota in ileal mucosa and *Lachnospiraceae* was significantly high, but *Helicobacter* and *Desulfovibro* were found to be significantly reduced [207]. In contrast, *Lactobacillaceae* and *Acidaminococcaceae* were increased but *Lachnospiraceae* and *Barnesiellaceae* were decreased on the ceca of FOS-water-supplemented broilers [208]. In addition to the modulation of gut microbiota, prebiotics also exhibit related immunomodulatory effects in relation to coccidiosis. Supplementing broilers with β-glucans in mixed *Eimeria* spp. infection resulted in reduced LS and downregulation of iNOS in the jejunum at day 10 after infection (D10), whereas upregulation in the ileum at D14, downregulation of mucin-2 at D14 and subsequent upregulation at D21, and downregulation of IFN-γ in the duodenum and ileum [187]. Dietary supplementation with MOS enhanced host resistance to coccidiosis through both performance- and immune-mediated mechanisms. In naturally *Eimeria*-exposed broilers, dietary supplementation with MOS at 0.05% in the form of yeast cell wall, improved BWG and FCR, reduced OPG, and enhanced both cell-mediated and humoral immune responses. Cell mediated immunity was evidenced by increased T-cell response assessed through basophilic hypersensitivity test, while humoral immunity was reflected by elevated Newcastle disease virus-specific antibody titers following concurrent vaccination and increased intestinal secretory IgA levels [188]. In coccidiosis vaccination-*Eimeria* challenge model, phased MOS supplementation (2 kg/tonne starter, 1 kg/tonne grower, 0.5 kg/tonne finisher) improved FCR in broilers, supporting its role as an effective adjunct to coccidia vaccination programs [189]. Additional mechanistic insight has emerged from studies using yeast-derived, nucleotide-rich prebiotics. In unchallenged broilers, such supplementation at 500 mg/kg improved feed efficiency but was associated with shortened villus height and increased SCFA concentration, indicating altered nutrient utilization and microbial fermentation patterns [190]. Notably, following *Eimeria* challenge, the same nucleotide-rich yeast extracts exerted context-dependent benefits, including improved BWG, restoration of jejunal villus height, increased expression of cationic amino tranporter-1 (CAT-1), modulation of cecal SCFA profiles and cecal pH, as well as increased immune organ development (e.g., bursa weight at day 35), collectively supporting enhanced epithelial recovery, nutrient transport, and immune competence under infection pressure [190,191]. Consistent with these findings, recent work using sugarcane molasses (1 mL/L) demonstrated a significant mitigation of ET induced performance losses, as evidenced by improved FCR and BWG, together with marked reductions in OPG. These improvements coincided with normalization of oxidative status in infected birds, reflected by decreased MDA levels and SOD activity, indicating attenuation of infection-associated oxidative stress [192].

### 4.2. Probiotics

Probiotics are defined by the World Health Organization and Food and Agricultural Organization as “live microorganisms which, when administered in adequate amounts, confer a health benefit on the host”. Probiotics, when given in sufficient amount, exert their function in supporting the host by improving gut barrier integrity, modulating immune response, synthesizing neurotransmitters and competitively excluding pathogens [209]. In broilers, a mixture of *Bacillus subtilis* and *Saccharomyces cerevisiae* improved BW and immune organs (e.g., the bursa of Fabricius and thymus) in addition to improved intestinal morphometry and tight junction mRNA expressions (e.g., OCLN, CLDN2, CLDN3), as well as inflammatory cytokines (e.g., IL-6, TNF-α, IL-10, TGF-β) [210]. The probiotic effect on production parameters is further corroborated in a different study with the spores of three *Bacillus bubtilis* strain in broilers [206]. In coccidiosis, *Lactobacillus plantarum* supplementation has been shown to mitigate infection-related effects while improving both cell-mediated and humoral-mediated immune responses, downregulating SOD and CAT expression and upregulating ZO-1 expression [193]. Similarly, dietary inclusion of *Saccharomyces cerevisiae* has demonstrated protective effects against ET infection [192].

### 4.3. Synbiotics

These are formulations that combine beneficial microbe probiotics with specific substrates used by host microorganisms (prebiotics) in different proportions to produce synergistic effects that improve host health [211]. In broilers, synbiotic supplementation can significantly improve overall growth performance, gut structure, dressing yield, feed efficiency, immune response, and intestinal microbiota [212,213,214]. A recent report showed that administering a synbiotic formulation with a combination of feed and water or water only has superior effects than when administered with feed alone [215]. In a mixed coccidia infection, a combination of different probiotics and inulin (PoultryStar^®^) was very effective against coccidiosis infection in promoting growth parameters in infected chickens, improving intestinal morphology, and modulating gut microbiota. Similarly, this multi-strain probiotic was also found to have more beneficial effects when used complementary with the coccidiosis vaccine [194]. Supplementation with specific synbiotics blends such as combinations of *Enterococcus Faecium*, *Lactobacillus* spp., *Pedicoccus acidilactici* and substrates such as dextrose, maltodextrin, inulin, oligofructose, and FOS (e.g., En-florax^®^, Biomin^®^IMBO) has shown beneficial effects in broilers challenged with EA and ET, including improved gut health and reduced infection severity [192,195]. Conversely, a recent report on synbiotic and mixed *Eimeria* spp. infection found no significant effects or inferior effects, suggesting the need for evaluation of these additives [216,217].

### 4.4. Postbiotics

These are preparations of inanimate microorganisms and/or their components (e.g., that confer benefits on the host [218]. In poultry, supplementation with postbiotics was related to improved immunity, growth performance, improved tibia health and reduced mortality [219]. Coccidia infection is a major predisposing factor for necrotic enteritis (NE), as epithelial damage, mucin hypersecretion, and microbiota disruption create an intestinal environment that favors *Clostridium perfringens* proliferation. Accordingly, successful experimental NE models involve sequential or combined challenge with *Eimeria* spp., and *C. perfringens* [220]. Within this context, a recent report demonstrated the potential of in ovo and drinking water supplementation of postbiotics to improve intestinal villi and crypt structures, increase the expression of mucin-2 and olfactomedin-4, and reduce inflammatory cytokines (TNF-α, iNOS, IFN-γ, IL-10). In the same study, they found that NE-challenged, postbiotic-supplemented broilers have higher mRNA expression of zonula occludens-1 and TNF-α in the jejunum and iNOS in the cecal tonsils compared to infected/untreated controls [196].

### 4.5. Metabolites

Microbial metabolites, including short chain fatty acids (SCFAs) such as acetate, butyrate and propionate, and medium chain fatty acids (MCFAs) such as caproic, caprylic, capric and lauric, are compounds produced during microbial metabolism that function as critical signaling molecules, energy substrates, and regulatory agents [221,222]. These molecules also participate in the host’s physiology, nutrient absorption, and immune development, and maintain gut barrier integrity and defense mechanisms against pathogens [223]. In coccidiosis, the action of SCFAs is through an indirect host-mediated mechanism that improves the overall disease tolerance of birds to infection, and the pathological consequences [224]. In mixed *Eimeria* spp. infection, supplementation with a butyric and valeric glyceride blend significantly improved production parameters and reduced OPG and LS in infected broilers [197]. Similar positive results were observed in EM-infected butyric acid glyceride supplemented broilers [198]. In an NE model, deoxycholic acid (DCA) and butyrate synergistically improved NE resistance [199].

### 4.6. Precision Biotics (BP)

These are an emerging category of non-antibiotic microbiome metabolic modulators. They are carbohydrates with glycosidic linkages (e.g., chemically defined glycans) that are engineered to steer gut microbial metabolism and safeguard the gut environment [225]. In addition to gut health, PBs are also known to exert beneficial effects on the environment by controlling nitrogen metabolism and protein utilization by the intestinal microbiome, thereby influencing nitrogen excretion and emissions [225,226]. In broilers, a PB-supplemented diet significantly improved the BWG and FCR of broilers, activated microbial protein metabolism, shifted microbiome metabolic function to nitrogen utilization pathways, increased the abundance of genes linked to beneficial protein metabolism, and improved litter and welfare in birds, as evidenced by reduced footpad lesions [226,227]. In a coccidiosis challenge model, PB supplementation improved FCR and ileal digestibility of dry matter, nitrogen and amino acids, and lowered litter ammonia levels [200]. Another study with mixed *Eimeria* spp. reported similar performance gains, in addition to improved LS and intestinal morphology, reduced IL-1β expression and increased expression of cell cycling gene markers (CCNA2 and CDK2) [201]. However, evidence on the use of PBs specific to *Eimeria* models remains limited to a number of trials with heterogeneity (e.g., glycan formulation, dosing, and challenge designs). The long-term field durability, synergy with other control measures and cost-benefit at scale still require further investigation.

### 4.7. Combined Strategies

Combining feed enzymes with direct-fed microbials (DFM or probiotics) represents another innovative strategy to improve nutrient utilization and gut health to increase a bird’s protection from coccidiosis. Indigestible dietary components can exacerbate coccidial pathology by fostering dysbiosis; therefore, exogenous enzymes (such as xylanases, amylases, and proteases) help to reduce the substrate for harmful microbes and improve the overall gut environment [228]. When used along with probiotics, there is a dual benefit: enzymes improve digestibility and reduce irritation to the gut lining, and probiotics competitively inhibit pathogenic microbes and modulate immunity [228,229]. Multi-enzyme-probiotic supplementation has been previously reported to improve FCR and BWG independently to antibiotic growth promoter (AGP) and can ameliorate coccidia infection by reducing plasma acute proteins, downregulating expression of inflammatory markers IL-6 and IL1β in the duodenum of *Eimeria*-infected chickens [202,230]. Supplementation with benzoic acid with EO compounds (Crina^®^ Poultry Plus) significantly improved FCR and BWG in supplemented broilers and alleviated EA-related LS [203]. In NE infection, a synbiotic-EO formulation reduced mortality at days 0–14, improved average daily gains (ADG), reduced LS in the duodenum but not in the jejunum and ileum, upregulated mRNA expression of CLDN-3, and reduced IFN-γ, IL-10, and sIgA compared to the non-supplemented group [204]. Together, this multipronged approach exemplifies how optimizing gut function can translate to improved tolerance or resistance to coccidiosis. It addresses not only the parasite itself but also the secondary factors (dysbiosis, inflammation) that affect the severity of coccidia disease.

Collectively, despite strong experimental support, microbiome- and gut-modulator-based interventions face several translational limitations. Efficacy is highly context-dependent, varying with baseline microbiota, diet composition, bird age, and *Eimeria* spp. challenge intensity which complicates standardization and reproducibility. Moreover, strain- and formulation-specific effects, incomplete resolution linking microbiome shifts to anticoccidial outcomes, and limited dose and timing optimization restrict cross-study comparability. Finally, robust data on long-term field durability, scalability, and cost-effectiveness under commercial settings remain scarce.

## 5. Host-Directed Immunological and Biotechnological Approaches

Several innovative strategies in addition to phytochemicals and microbial modulators are currently being explored to provide precision-targeted protection against coccidiosis. Among these are immunological modulators, nanotechnology-based approaches and delivery systems (Table 4).

### 5.1. Host-Directed Immunotherapies

Immunotherapies encompass a broad range of strategies that harness or modulate the host’s immune response to prevent or control diseases. Although several phytogenics and microbiome modulating agents mentioned above have been shown to improve mucosal immunity by increasing secretory IgA, improving epithelial integrity, and regulating cytokine expression, their immunomodulatory effects are largely secondary to gut health optimization [231,232]. In contrast, host-targeted immunotherapies represent an emerging frontier in poultry aimed at activating specific immune responses. These approaches include the use of host defense peptides (HDPs), hyperimmune egg yolk-antibodies (IgY), recombinant vaccines, and transgenic vaccine technologies.

#### 5.1.1. Host Defense Peptides (HDPs)

HDPs, also known as antimicrobial peptides (AMPs), are cationic, amphipathic effector molecules of the innate immune system that have extensive antimicrobial activity, low risk of resistance and potent immunomodulatory properties [233]. HDPs serves as a bridge between innate and adaptive immunity and are widely being explored in the pharmaceutical and food industries and biomedical fields for their antimicrobial and anti-inflammatory, wound healing and immunotherapeutic potential, including as novel immune adjuvants [234]. In avian species, identified HDPs include NK-lysin (cNK-lysin), liver-expressed antimicrobial peptide 2 (LEAP2), cathelicidins (CATH1-3 and CATH-B1), avian β-defensins (AvBD1-14), and ovodefensins (gallin 1-3), all with known broad antimicrobial activities and potent immunomodulation on mucosal surfaces [233,235,236,237]. These HDPs were found to have direct activity that disrupts microbial/parasite membranes, improves epithelial barrier function, and modulates immune responses by regulating cytokine production and immune cell recruitment, highlighting their potential as a host-directed tool for controlling enteric pathogens such as *Eimeria* spp. [236,237]. cNK-lysin evidently exhibits anticoccidial activity by directly reducing the viability of EN sporozoites and inducing the expression of inflammatory cytokines (IL-1β and CXCLi2) in chicken macrophages [238]. cNK-lysin consistently showed direct cytotoxicity against EA and ET sporozoites in vitro and conferred in vivo protection to EA-infected broilers when given as a dietary supplement [239,240]. Chicken β-defensin-1 similarly exhibits bonafide anticoccidial activity in broilers by significantly lowering OPG in ET-challenge broilers [241]. Although HDP-based adjuvants are being extensively explored in humans, the evidence for avian HDPs as vaccine adjuvants remains limited. The results of cNK-2 adjuvanted *Eimeria tenella* elongation factor-1α (EF-1α) recombinant vaccine far underperformed its recombinant IL-7-adjuvanted counterpart in a coccidiosis model, underscoring the context-dependent efficacy of HDPs as adjuvants and the critical roles of optimized formulation and delivery [240].

**Table 4:** List of advanced innovative control strategies for coccidiosis with their anticoccidial effects in broilers.

Control Strategy/ Approach	Representative Sources/Products	Main Bioactive Components	Effects on Coccidiosis	References
Host-directed immunotherapies	Host defense peptides (HDPs)	NK-Lysin	**EA:** direct effect on sporozoite viability, reduce LS, improve BW; **EN:** direct effect to sporozoite viability and induce expression of IL-1β and CXCLi2; **ET:** direct effect on sporozoite viability	[238,239]
		β-defensin-1	ET: reduce OPG	[241]
	Hyperimmune Egg Yolk antibodies	IgY against *Eimeria* antigens	**EA:** reduced OPG and improve BWG; **EM:** reduce LS, improve BW, reduced OPG; **ET:** reduce LS, improve BW, reduced OPG in feces and cecal tissue	[242,243,244]
	Recombinant immunobiologics	pcDNA3-1E vaccine adjuvanted with IL-1β, IL-2, IL-8, IL-15, IFN-α, IFN-γ TGF-β4 and lymphotactin	**EA**: improve BWG (IFN-α (1 μg) or 10 μg of lymphotactin), reduce parasite replication (10 μg of IL-8, lymphotactin, IFN-γ, IL-15, TGF-β4, or IL-1β), increase CD3+ cells (IL-8 or IL-15)	[245]
		recombinant EN glutathione peroxidase (EnGPX)	**EN:** robust humoral response, reduce LS, OPG, and sporulation rate	[246]
		recombinant chicken IL-7 (rchIL-7)/recombinant *Eimeria* elongation factor-1α (rEF-1α) + chicken NK-lysin peptide 2 (cNK-2)	**EM:** high levels of serum IL-7, improved BWG, reduced OPG, reduced LS, reduced expression of cytokines (IL-6, IL-10, IFN-γ), increase OCLN	[240]
		recombinant EtMIF (Macrophage migration inhibitory factor)	**ET:** reduced LS and OPG, improve ACI and elevate serum antibodies and cytokines (i.e., IL-1, IL-8, IFN-γ, and TNF-α)	[247]
Engineered biotherapeutic products	*Eimeria*-vectored vaccines (transgenic vaccines)	transgenic *E. tenella* expressing *E. maxima* AMA1 and IMP1	**EM:** reduce parasite replication in the intestine, low LS, intermediate levels of IL-10 and IFN-γ	[248]
		Transgenic *E. tenella* expressing *E. Maxima* profilin	**ET:** enhance parasite-specific cell-mediated immunity, enhanced immunoprotection by reduced LS, solid protective immunity with OPG clearance at 28 d (ET dose at 10,000) and reduce OPG at high dose ET infection (100,000), gut modulation; **EM:** no significant reduction in OPG	[249]
		Transgenic *E. necatrix* expressing IL-1β	**EN:** reduce OPG, increase CLDN-1 and Avian β-defensin-1	[250]
		Transgenic *E. mitis* expressing chicken IL-2	**E. mi:** higher cellular immune response, reduce OPG	[251]
	Probiotic vectored vaccines	Recombinant *Lactococcus lactis* expressing chicken IL-4 and IL-2 fusion protein	**Mix** ***Eimeria*** **spp.:** synergistic with coccidia vaccine to improve BW, reduced OPG and LS, high ACI	[252]
		*Saccharomyces cerevisiae* expressing EtAMA1 (Apical Membrane Antigen 1), EtIMP1 (Immune Mapped Protein 1) and EtMIC3 (repeat 3 from Microneme Protein)	**ET:** reduced parasite replication in ceca, improved BWG in Cobb500 broilers (D21-31), reduce LS in Hy-line brown layers	[253]
		*Bacillus subtilis* (*B. subtilis*) expressing cNK-2	**EA:** improved growth performance, reduced OPG, enhanced gut integrity (OCLN, ZO-1, JAM-2, MUC-2)	[254]
Nanotechnology and delivery systems	Encapsulation of Essential oils	Eugenol Nanoemulsion	**Mix** ***Eimeria*** **spp.:** reduce OPG, improve daily feed intake, daily weight gain and FCR, increase levels of serum globulins, glutathione S-transferease, glutathione peroxide	[255]
	Nanoparticle formulation	nanocurcumin	**Mix** ***Eimeria*** **spp.:** increase BW and carcass weight	[256]
		*Azadirachta indica* ethosomal nanovesicle	**ET:** improve BW, and serum reduced glutathione (GSH), decreased feed intake, FCR, OPG, MDA, and NO serum levels, reduced protein expression of IL-1β, IL-6, BAX, and caspase-3 in cecal tissue, mitigated cecal tissue damage	[77]
		Green synthesis of iron-oxide nanoparticle from *Ficus racemosa*	**ET:** reduced OPG and LS, improve BWG and FCR, significant tissue recovery post infection, no mortality	[257]
		*Lactobacillus casei* EE12-mediated zinc nanoparticles (Lc-ZnNPs)	**ET:** improve BWG and FCR; reduced MDA; enhance SOD, CAT and GPx; downregulate Mucin-1, OCCU, IL-6, IL-1β, Bcl-2-associated protein x and casp-3; reduce OPG and LS; reduce total bacterial count, total yeasts and molds, and *Salmonella* count; enhanced lactic acid bacteria count	[258]
		Recombinant EM IMP1 antigen nanovaccine	**EM:** improved WG in battery studies, partial protection in floor pen studies	[259]
		PLGA encapsulated recombinant Emi 1a protein	**E. mi:** upregulate secretion of antibodies (IgY) and cytokines (IFN-γ, IL-4, IL-6, IL-10, IL-17) and proportions of CD4+ and CD8+ lymphocytes, improve growth performance, reduce oocysts	[260]
Systems biology/Multi-omics pipeline discovery platform	In silico and in vitro identification of novel vaccine or drug targets through genomic, transcriptomic, proteomic and metabolomic integration	in silico selection of dense granule protein (DGP) vaccine candidates	**recombinant EtGRA9 + ET:** improved BWG, reduced OPG	[261]
		multiepitope vaccine encoding four *Eimeria* epitopes in PLGA nanospheres	**NSLC-PLGA (Epitopes of EN: NA4, ET:** SAG1, EA: LDH, EM: CDPK) + EA/EM/EN/ET: potentiate humoral (IgY) and cellular (CD4+/CD8+ T-cell) immune responses, reduce OPG, improved growth coefficients, cross protection across *Eimeria* spp. challenge	[262]
		CRISPR-CAS9 based RNA drugs	**sgRNAs/Cas9 mRNA + ET:** reduced oocysts sporulation and sporozoite viability in vitro, reduce pathogenicity of ET in vivo	[263]

ACI, anticoccidial index; BW, body weight; BWG, body weight gain; CAT, cationic amino acid transporter-1; CLDN, claudin; FCR, feed conversion ratio; EA, *Eimeria acervulina*; EM, *E. Maxima*; E. mi, *E. mitis*; EN, *E. necatrix*; ET, *E. tenella*; IL, interleukin; JAM, junction adhesion molecule; LS, lesion score; MUC, mucin; MDA, malondialdehyde; NO, nitric oxide; OCLN, occludin; OPG, oocyst per gram; SOD, superoxide dismutase; ZO-1, zonula occludens-1.

#### 5.1.2. Hyperimmune IgY

Immunoglobulin Y (IgY), the avian counterpart of mammalian IgG, is the primary antibody involved in humoral immune defense in birds [264]. This antibody is mainly found in the serum and egg yolk, and is naturally transferred from hens to embryo to confer maternal immunity during early life [264,265]. Using this physiological transfer, hens can be hyperimmunized with specific *Eimeria* antigens to produce specific IgY that accumulates in egg yolks. This approach allows large-scale noninvasive antibody production and offers a residue-free and animal welfare-friendly platform for passive immunization in poultry as an alternative to conventional chemotherapeutics [264]. The proposed mechanisms of IgY in pathogen activity include agglutination, adhesion blockade, and opsonization, followed by phagocytosis and toxin neutralization [266]. In coccidiosis, IgY is thought to exert its anticoccidial activity through antigen-antibody interactions with merozoites and sporozoites, blocking adhesion to and invasion into epithelial cells and thereby limiting replication and intestinal injury, although the precise mechanism remains to be fully elucidated [267]. Experimental evidence substantiates the protective potential of hyperimmune IgY against major *Eimeria* spp. In broilers challenged with EA, dietary inclusion of Supracox^®^ (egg yolk powder with hyperimmune IgY) at 10 or 20% significantly improved BWG and reduced OPG [242]. Comparable protection against ET and EM, where IgY supplementation mitigated intestinal lesions and weight loss relative to controls in a basal diet, was also observed [243]. A multivalent IgY formulation targeting five *Eimeria* spp. (1% inclusion rate) improved growth performance, eliminated mortality, and significantly lowered lesion severity following ET challenge [244]. Supplementation with 120 mg/kg in specific pathogen-free (SPF) Leghorn chicks showed a significant anticoccidial effect in ET-infected birds, including OPG reduction both in feces and cecal tissue, suggesting that IgY-based immunotherapy not only reduces parasite invasion and replication but also modulates intestinal homeostasis and inflammation [267].

#### 5.1.3. Recombinant Immunobiologics

These integrate parasite-directed antigens with host-directed modulators to amplify mucosal/cellular immunity against *Eimeria* as a sustainable non-antibiotic strategy. Early proof of concept using pcDNA3-1E vaccines co-adjuvanted with cytokine genes (e.g., IL-1β, IL-2, IL-8, IL-15, IFN-α, IFN-γ TGF-β4 and lymphotactin) demonstrated strong immunopotentiation in an EA infection model, where IFN-α (1 μg) or lymphotactin (10 μg) protected White Leghorn chickens from weight loss, 10 µg plasmids of IL-8, lymphotactin, IFN-γ, IL-15, TGF-β4, or IL-1β significantly reduced OPG, and IL-8 and IL-15 elevated CD3+ cells in duodenum intraepithelial lymphocytes [245]. Similarly, recombinant EN glutathione peroxidase (EnGPX) induced robust humoral responses, lowered LS and OPG, and diminished oocysts sporulation, suggesting an additional antioxidant-mediated protective mechanism [246]. Contemporary advances in cytokine-enhanced recombinant protein vaccines further validate the immunobiologic framework. In EM infection, the combined administration of recombinant chicken IL-7 (rchIL-7) or chicken NK-lysin peptide-2 (cNK-2) with recombinant *Eimeria* elongation factor-1α (rEF-1α) significantly improved BWG, reduced OPG and LS, downregulated proinflammatory cytokines (e.g., IL-6, IL-10, IFN-γ), and upregulated occluding (OCLN) expression, indicating improved barrier function and immune homeostasis [240]. Likewise, recombinant EtMIF (macrophage migration inhibitory factor, rEtMIF) elicited broad immunoprotective effects during ET infection by significantly reducing LS and OPG in immunized chickens, elevating serum IL-1, L-8, IFN-γ, and TNF-α, and improving the anticoccidial index (ACI) [247]. Together, these recombinant biologics show consistent improvements of the negative effects of coccidiosis in chickens, reinforcing their value as an antimicrobial-independent, immune-centric intervention for control.

### 5.2. Engineered Biotherapeutic Products

These products represent an advanced class of biologics that use a live microbial or protozoan vector to deliver immunoprotective antigens or immunomodulators directly to the gut, mimicking natural infection while avoiding drug residues or antimicrobial reliance [268]. Recently, transgenic *Eimeria* is gaining attention as an alternative strategy. The foundational concept for transgenic *Eimeria* platforms stems from studies on the biology of *Eimeria* sporozoites, which identifies the immunogenic potential of surface antigens such as apical membrane antigens (AMAs) and immune mapped protein-1 (IMP-1) as immunogenic determinants of host cell recognition and entry, making them prime targets for vectored expression [269]. Among *Eimeria*-vectored vaccines, transgenic ET expressing EM AMA-1 and IMP-1 have achieved significant cross-protection by reducing EM replication in the intestine, reducing LS and modulating cytokine profiles with intermediate IL-10 and IFN-γ levels [248]. Likewise, transgenic ET expressing EM profilin improved parasite-specific cell-mediated immunity, conferred solid protective immunity with oocyst clearance by 28 days at moderate challenge dose, reduced OPG and LS severity even at high infection levels, and modulated microbial diversity. However, the vaccine was only effective against ET challenge and not for EM, underscoring species/antigen matching constraints even when heterologous expression is possible [249]. Extending this approach, transgenic EN expressing IL-1β significantly reduced OPG and improved intestinal barrier function with increased CLDN-1 and avian β-defensin-1 expression, whereas transgenic *E. mitis* expressing chicken IL-2 improved cellular immune activation and decreased OPG [250,251].

Parallel efforts in probiotic-vectored systems demonstrate that food-grade microbes can act as safe and scalable antigen delivery vehicles. Recombinant *Lactococcus lactis* expressing chicken IL-4/IL-2 fusion protein showed synergistic protection during infection when co-administered with live coccidial vaccines, improving BWG, reducing OPG and LS, and yielding a high ACI in broilers [252]. Similarly*, Saccharomyces cerevisiae* yeast expressing three ET antigens (EtAMA1: Apical Membrane Antigen 1, EtIMP1: Immune Mapped Protein 1, and EtMIC3: repeat 3 from Microneme Protein) reduced parasite replication and LS in Hy-Line Brown layer chickens and improved BWG in Cobb500 broilers [253]. A complementary probiotic vector using spore-forming *Bacillus subtilis* displaying cNK-lysin also conferred protective effects in EA-challenged broilers. Oral delivery of the engineered probiotic reduced OPG shedding, improved growth performance and upregulated the expression of epithelial barrier genes (OCLN, ZO-1, JAM-2, MUC-2) [254]. Together, these platforms exemplify a new generation of engineered immunobiologics that deliver multi-stage *Eimeria* antigens and cytokine payloads in vivo and stimulate broad mucosal immunity without reliance on chemotherapeutics. However, future studies should focus on vector optimization and multi-antigen co-expression, standardized oral formulations, and field trials in mixed-species exposure to confirm durability and scalability.

### 5.3. Nanotechnology and Delivery Systems

Effective control of diseases increasingly depends not only on what is delivered but on how it is delivered. Many promising natural products and biologics are constrained by their poor solubility, limited biological distribution and rapid metabolic [30,270]. Modern drug delivery platforms address these bottlenecks by protecting cargos through the upper gastrointestinal tract, increasing epithelial residence and uptake, enabling controlled release at the infection site with high specificity and sensitivity, and targeted precision therapy, thereby improving performance outcomes while minimizing off-target interactions [271]. In this paradigm, nanotechnology has emerged as a practical mucosa-targeted solution for parasitic disease management including coccidia [260,272].

Nanoencapsulation of EOs in carrier systems, including lipid-based (e.g., nanoemulsions, liposomes, solid lipid nanoparticles and nanostructured lipid carriers), polymer-based (e.g., chitosan), or inorganic-based (e.g., metal and metal oxide NPs), represent a key innovation for poultry health that overcomes the inherent instability of volatile phytochemicals. This strategy protects the labile EOs from oxidative and enzymatic degradation and ensures sustained release and prolonged intestinal residence time, which collectively improves their bioavailability, gut adsorption and ultimately their efficacy [30]. In broilers challenged with mixed *Eimeria* spp., dietary supplementation of clove essential oil (CEO), eugenol (EUG), and their nanoemulsions (Nano-CEO, Nano-EUG) demonstrated that Nano-EUG produced the most pronounced improvements by significantly reducing OPG, improving growth performance parameters, restoring serum globulins, and elevating glutathione S-transferase and peroxidase activities, indicating potent antioxidant and immunoprotective effects [255].

In addition to EOs, nanoparticle formulations provide a versatile platform for delivering natural or bioengineered compounds with high stability and biological precision. For instance, nanocurcumin supplementation improved BWG and carcass yield in broilers infected with mixed *Eimeria* spp., highlighting the promising antioxidant potential of nanocurcumin and its positive effect on chicken meat quality [256]. Similarly, an ethosomal nanovesicle formulation of *Azadirachta indica* leaf extract protected ET-infected broilers by improving BWG, reducing feed intake and feed conversion ratio (FCR), and decreasing OPG, malondialdehyde (MDA), and nitric (NO) levels while downregulating IL-1β, IL-6, BAX, and caspase-3 expression in cecal tissues, effectively mitigating inflammation and apoptosis [77].

Green-synthesized metallic nanoparticles have also shown promise as a biocompatible, eco-friendly alternative to conventional chemotherapeutics. The use of the aqueous lead extract of *Ficus racemose*, iron oxide nanoparticles (IONSPs) fabricated with green synthesis significantly reduced OPG and LS, improved BWG and FCR, facilitated tissue recovery, and prevented mortality in ET-infected broilers. Their antiparasitic efficacy was confirmed at a dose of 15 mg/kg [257]. A more recent report using *Lactobacillus casei* EE12-mediated zinc nanoparticles (Lc-ZnNPs, 41 nm) demonstrated marked reduced OPG and LS, improved antioxidant enzyme activity (SOD, CAT and GPx), and downregulated intestinal and hepatic inflammatory and apoptotic mediators (Mucin-1, OCCU, IL-6, IL-1β, Bcl-2-associated protein x and casp-3) in ET-infected broilers. In the same study, Lc-ZnNPs also modulated the gut microbiota by lowering total bacterial, yeast, mold and *Salmonella* counts while increasing lactic acid bacteria populations to collectively improve the growth performance and intestinal health of the broilers [258].

Recombinant nanovaccines further extend nanotechnology into targeted immunoprophylaxis against *Eimeria* spp. In EM, a recombinant IMP1 antigen nanovaccine improved weight gain and full protection in battery challenge trials while conferring partial protection in floor-pen trials, validating its feasibility as a safer, subunit-based vaccine alternative [259]. Meanwhile, PLGA-encapsulated recombinant *E. mitis* 1A protein nanoparticles significantly upregulated antibodies (IgY) and cytokines (IFN-γ, IL-4, IL-6, IL-10, IL-17), expanded CD4+ and CD8+ lymphocyte populations, and reduced oocyst shedding, correlating with improved growth and mucosal protection [260]. Together, these studies demonstrate that nanotechnology-enabled systems, from phytogenic encapsulation to metallic and recombinant nanovaccines, can synergistically improve parasite suppression, mucosal immunity and oxidative balance while reducing reliance on conventional anticoccidials. However, future effort should focus on optimizing particle characterization, biocompatibility, release kinetics, safety, and field validation to advance nanotherapeutics as precision and residue-free tools for control.

Despite strong proof-of-concept, host-directed immunotherapies and biotechnological platforms remain constrained by several translational bottlenecks. For immunomodulators such HDPs and IgY, efficacy is highly dependent on formulation, dosing, stability and delivery route with limited data and large-scale production, bioavailability and consistency under field conditions. Recombinant immunobiologics and engineered biotherapeutics face additional challenges related to antigen selection, species- and strain-specific protection, vector optimization and regulatory complexity particularly for transgenic or genetically modified platforms. Nanotechnology-based delivery systems markedly enhance bioavailability and efficacy of compounds; however, they are limited by incomplete characterization of long-term safety, tissue accumulation, cost-effectiveness and scalability in commercial poultry systems. Collectively, the lack of standardized formulations, pharmacokinetic data, multi-species challenge models and large-scale field validation currently restricts the transition of these strategies to routine industry application.

## 6. Precision and Omics-Guided Biotherapeutic Platforms

High-resolution genomics, transcriptomics, proteomics, metabolomics, and metagenomics are redefining anticoccidial discovery by mapping host–parasite–microbiome interactions to actionable nodes. Integrated analyses of infected intestinal tissues delineate coordinated host programs in innate/mucosal immunity, metabolism, and epithelial repair, while parasite stage-resolved datasets highlight invasion and virulence factors as tractable antigens or drug targets [273,274]. Likewise, metabolic and microbiome analyses delineate how infection reshapes gut microbial communities and metabolic fluxes and provide a blueprint for next-generation probiotics, postbiotics and dietary immunomodulators aimed at restoring colonization resistance and intestinal homeostasis [274,275]. Together, these multi-omics frameworks enable rational target discovery and the precision formulation of biologics, vaccines, and metabolic co-therapeutic interventions.

Recent applications exemplify the translational potential of these platforms in poultry coccidiosis research. Using comparative genomics and in silico analysis, Sanchez-Arsuaga et al., (2025) identified a limited repertoire of dense granule proteins (EtGRA9, EtGRA12a, and EtGRA12b) in ET relative to *Toxoplasma gondii* [261]. Recombinant EtGRA9 vaccination resulted in significant improvements in weight gain and reduced parasite load upon challenge, validating GRAs as promising vaccine candidates [261,262]. Similarly, reverse vaccinology and computation epitope mapping guided the design of a multiepitope antigen (NSLC) encoding epitopes from four *Eimeria* spp. (EN: NA4, ET: SAG1, EA: LDH, EM: CDPK). When encapsulated in PLGA nanospheres, NSLC elicited robust humoral (IgY) and cellular (CD4+/CD8+ T-cell) immunity, reduced oocyst shedding, and improved growth. Moreover, intramucosal delivery of the optimized NSLC-PLGA (300 µg) vaccine was the most efficient approach to induce broad cross-protection against challenges with EA, EM, EN and ET, demonstrating the feasibility of cross-protective nanovaccines [262]. In a complementary approach, CRISPR-Cas9 based on nucleic acid therapeutics targeting ET ITS and 5.8S rDNA sequences produced marked antiparasitic activity in vitro by suppressing oocyst sporulation and diminishing sporozoite viability, and treated parasites had lower lesion and oocysts scores in vivo [263]. Together, these studies illustrate how multi-omics-driven target discovery, in silico design and genome editing tools are redefining the frontier for coccidiosis control. However, despite their potential and novelty, current evidence is still preliminary, and most studies are limited to experimental validation under controlled settings. Future work should address scalability, delivery feasibility, cross-strain efficacy, and economic viability so that omics-guided and gene-based interventions can advance from conceptual work to practical, field-ready anticoccidial solutions.

## 7. Knowledge Gaps and Future Research Priorities

Despite substantial advances in non-antibiotic coccidiosis control, several critical knowledge gaps constrain the transition of these strategies from experimental promise to field adaptation.

1. Lack of standardization in phytochemicals and botanicals: Chemical compositions vary widely between batches and sources, limiting reproducibility and making it difficult to identify which active constituents drive efficacy.
2. Insufficient pharmacokinetic and delivery data: Key compounds such as curcumin, allicin, and essential oil constituents, information on absorption, metabolism, tissue distribution, and optimal delivery systems remain sparse, hindering dose optimization and field translation.
3. Species and strain coverage gaps in *Eimeria* models: Research is disproportionately focused on ET and EA, whereas EM, EN, EP, and mixed infections are substantially underrepresented.
4. Limited insight into nutrient–parasite–immune interactions: How dietary protein density, amino acid balance, fat source, fiber level, and trace mineral supplementation modulate host resilience, immune activation and *Eimeria* pathobiology is not fully understood.
5. Incomplete understanding of microbiome-mediated mechanisms: Although biotic and microbial metabolites show efficacy, the causal relationship between microbiome restructuring, SCFA/MCFA dynamics, mucosal immunity and anticoccidial outcomes remains unresolved.
6. Early-stage development of innovative technologies: Omics-guided vaccines, nanovaccines, genome-editing therapeutics, and host defense peptides are still largely at the proof-of-concept stage, and there is minimal data on long-term safety, scalability and field performance.
7. Absence of systematic factorial and combination studies: Potential synergies and combination between domain approaches remain unexplored, limiting the rational design of integrated, multi-modal anticoccidial programs.

Given these gaps, future progress in sustainable coccidiosis control will depend on a more systematic and precision-oriented development pipeline. Advancing the efficacy and consistency of promising phytogenic anticoccidials will require more rigorous characterization of their active components and systematic testing of defined combinations. The integration of metabolomic profiling and data-driven formulation approaches offers a practical path to improving standardization and identifying synergistic mixtures, although further validation in controlled and field settings is still needed. Similarly, advances in poultry microbiome science provide a realistic opportunity to design next-generation biotic components that support epithelial repair, immune resilience and colonization resistance during *Eimeria* infection achieved through strategic selection, validation, and combinations of strains with reproducible functional outcomes. Innovations in delivery technologies also offer a promising practical means to stabilize and improve the delivery of compounds, positioning them for scalable application in poultry. Concurrently, multi-omics tools will enable rational antigen discovery and can accelerate the development of cross-protective vaccines and adjunct immunotherapies. Although genome editing and nucleic-acid based therapeutics are still at an early stage for parasitic diseases, their strategic incorporation into research pipelines may clarify molecular targets that hold genuine translational potential. Ultimately, multi-site field trials, dose optimization, and economic analyses will be needed to determine which combinations provide consistent, cost-effective protection. As these domains mature, an integrated, multimodal framework built on non-antibiotic strategies, precision microbiome modulation, and omics-guided innovation offers a realistic pathway to more sustainable control.

## 8. Conclusions

Non-antibiotic strategies, including phytochemicals, precision nutrition and minerals, microbiome-directed biotics, and emerging biotechnologies, offer credible and increasingly powerful alternatives for coccidiosis control. Together, these approaches demonstrate the capacity to reduce parasite burdens, protect mucosal integrity, and improve bird performance while supporting the global transition to antibiotic-free production. Importantly, the evidence synthesized in this review indicates that the greatest translational potential lies not in single interventions, but in rationally designed multi-modal strategies that combine parasite-directed suppression with host resilience, microbiome stabilization, and epithelial recovery. Phytogenic compounds and functional nutrients show promise as frontline modulators of parasite development and gut integrity, while microbiome-targeted interventions and adjuncts offer complementary mechanisms that enhance recovery and reduce disease recurrence.

However, widespread adoption remains limited by challenges in standardization, mechanistic activity, delivery efficiency, and field validation. It will be essential to overcome these barriers to translate experimental success into reliable commercial solutions. Ultimately, the integration of natural products with microbiome science and omics-informed innovation provides a realistic and future-ready pathway to sustainable, high-welfare, and antibiotic-independent coccidiosis management.

## Figures and Tables

**Table 2:** List of functional nutrition and mineral modulator control strategies for coccidiosis with their anticoccidial effects in broilers.

Control Strategy/ Approach	Strategy	Effects on Coccidiosis	References
Protein	High indigestible protein	**Mix** ***Eimeria*** **spp.:** reduce BWG, decreased ileal nitrogen digestibility, increased ileal indigestible nitrogen concentration, decreased goblet cell count, decreased expression of OCLN in ceca	[137]
	Low crude protein	**Mix** ***Eimeria*** **spp.:** improved ileal digestibility (in combination with threonine, arginine or glutamine); improved villus height (with threonine); reduced intestinal permeability and gene expression of AA transporters (with Arginine); decreased expression of CLDN-1 (with Threonine and glutamine)	[138]
Functional Amino Acid (AA)	Dietary AA density (i.e., digestible Lys)	**EA:** high AA improved BWG and feed efficiency; **Mix** *Eimeria* **spp.:** increased BWG, increased incidence of footpad lesion	[139,140]
	Mix AA (L-arginine, L-threonine and L-glutamine)	**Mix** ***Eimeria*** **spp.:** increased BWG, improve FCR, reduced LS in duodenum, not significant on OPG reduction, increased digestibility of the amino acid	[141]
	Arginine	**Mix** ***Eimeria*** **spp.:** increased villus height and villus height:crypt depth ratio, decreased LS	[142]
Carbohydrates	Insoluble fiber (Sunflower hulls)	**Mix** ***Eimeria*** **spp.:** improved BWG and FCR, reduced OPG and LS, increased intestinal morphology	[143]
Lipids	n-3 fatty acids (Fish oil)	**Mix** ***Eimeria*** **spp.:** reduced gastrointestinal permeability and LS	[144]
	n-3 fatty acids (Flaxseed and menhaden oil)	**ET:** reduced LS; **EM:** no significant effect on LS	[145]
Trace minerals	Zinc (Zn)	**NE (EM +** ***Clostridium perfringens*****):** increased LS, higher BWG, downregulated expression of IL-8, upregulated expression of IL-10; **Mix** ***Eimeria*** **spp.:** reduced OPG, reduced intestinal permeability, reduced LS in ceca	[146,147]
	Copper (Cu)	**ET**: reduced LS and oocyst index value; **Mix** ***Eimeria*** **spp.:** no significant effect on BWG, LS, intestinal permeability and OPG	[147,148]
	Manganese (Mn)	**EA:** improved weight gain (in combination with Cobalt)	[149]
	Multicomplex mineral	**EA:** reduced OPG, improved BWG	[150]
	Methionine with mineral blend (Zn, Cu, Mn)	**Mix** ***Eimeria*** **spp.:** improved FCR and cumulative performance index, downregulated expression of IL-17A in jejunum	[151]
	Advanced chelated form of Trace minerals (Cu, Zn, Se, Fe, Mn, Cr, I)	**Mix** ***Eimeria*** **spp.:** increased BWG and carcass yield; reduced serum alkaline phosphatase activity; reduced *E. coli* in the ileum; increased blood total antioxidant capacity, glutathione peroxidase, catalase and SOD; reduced MDA; increase expression of TGF-β; downregulated expression of IL-1β, IL-6 and IFN-γ; increased serum Fe, Zn, Mn, Cu levels	[152]
	Cu + Zn	**Mix** ***Eimeria*** **spp.:** reduced OPG, improved microscopic features in the jejunum, reduced LS	[153]

BWG, body weight gain; CLDN-1, claudin-1; FCR, feed conversion ratio; EA, *Eimeria acervulina*; EM, *E. Maxima*; ET, *E. Tenella*; IL, interleukin; IFN-γ, interferon-γ; LS, lesion score; MDA, malondialdehyde; NE, necrotic enteritis; OCLN, occludin; OPG, oocyst per gram; TGF-β, Transforming Growth Factor-β.

**Table 3:** List of microbiome and adjunct gut modulator control strategies for coccidiosis with their anticoccidial effects in broilers.

Control Strategy/ Approach	Representative Sources/Products	Main Bioactive Components	Effects on Coccidiosis	References
Prebiotics	Mannan oligosaccharides (MOS), β-glucans	non-digestible substrates	**MOS + Mix** ***Eimeria*** **spp.:** improve BWG and FCR, reduce OPG, increase cell mediated and humoral immune response, increase mucosal IgA, complementarily improved FCR with coccidia vaccine; **β-glucans + Mix** ***Eimeria*** **spp.:** reduce LS, downregulate iNOS (jejunum, D10), upregulate iNOS (ileum, D14), downregulate Mucin-2 (D14), upregulate Mucin-2 (D21), downregulate IFN-γ (duodenum and ileum)	[187,188,189]
	Nucleotide-rich yeast extract	non-digestible substrates	**Mix** ***Eimeria*** **spp.:** improve BWG and FCR, no effect on OPG and LS, improve CAT-1 in jejunum, increase villus height in jejunum, reduced SCFA increase pH in ceca digesta, increase bursa weight at d35, improve intestinal function	[190,191]
	Sugar cane molasses	non-digestible substrates	**ET:** reduce OPG and LS, improve FCR, decrease SOD, reduce concentration of MDA	[192]
Probiotics	*Lactobacillus*, *Bifidobacterium*, *Bacillus, Saccharomyces*	live microbes	***Lactobacillus plantarum*** **+ ET:** reduce fecal score and OPG, improve FCR, improve cell mediated and humoral immune response, reduce SOD and CAT expression, increase ZO-1 expression; ***Saccharomyces cerevisiae*** **+ ET:** reduce OPG and LS, improve FCR, decrease SOD, reduce concentration of MDA	[192,193]
Synbiotics	PoultryStar^®^ (*Enterococcus faecium*, *Bifidobacterium animalis*, *Lactobacillus salivarius*, Inulin)	Probiotic + prebiotic combination	**Mix** ***Eimeria*** **spp.:** improve BWG, reduce LS in ceca and ileum, reduce OPG, increase villus height in the duodenum, jejunum and ileum, high villus height to crypt depth ratio; higher lactic acid bacteria count ileum and ceca, lower coliforms in ileum; additive effects with coccidia vaccine in reducing LS and improving BW	[194]
	*Enterococcus Faecium* + Inulin	Probiotic + prebiotic combination	EA, ET: improve BW, OPG, and LS	[195]
	En-florax^®^ (dextrose, maltodextrin, inulin, oligofructose, fructo-oligosaccharides*, Enterococcus faecium, Lactobacillus casei, L. plantarum, Pedicoccus acidilactici*)	Probiotic + prebiotic combination	**ET:** reduce OPG and LS, improve FCR, decrease SOD, reduce concentration of MDA	[192]
Postbiotics	*S. cerevisiae*-based liquid postbiotic	microbial product component	**EM +** ***Clostridium perfringens*** **(Necrotic enteritis, NE):** upregulated expression of ZO-1 and TNF-α in jejunum, increase iNOS in cecal tonsil	[196]
Metabolites and acidifiers	Short Chain Fatty Acids, Medium Chain Fatty Acids	Butyrate, acetate, propionate, caproic, caprylic, capric and lauric	**Butyric and valeric glycerides + mix** ***Eimeria*** **spp. infection:** improve FCR, flock uniformity, reduce OPG, duodenal LS; **Butyric acid glycerides + EM:** reduced OPG, lesion score and mean number of developmental stages in lamina propria of jejunum, total cholesterol and total protein; improve BWG and FCR; **deoxycholic acid and butyrate + NE (EM** ***and Clostridium perfringens*****):** synergistic effect to induce multiple HDPs and CLDN-1 expression.	[197,198,199]
Precision Biotic (PB)	Glycan based PB	Oligosaccharide consortia	**Mix** ***Eimeria*** **spp.:** improved FCR and ileal digestibility of DM, N and amino acids; reduce LS, improved intestinal morphology, downregulate IL-1β, upregulate CCNA2 and CDK2	[200,201]
enzyme blends + direct fed microbial	Xylanase, Amylase, Protease, three *Bacillus* spp.	Enzyme blends and probiotics	**Mix** ***Eimeria*** **spp.:** improved BWG, reduced FCR, reduced plasma acute phase protein, downregulate expression of IL-6 and IL-1β in duodenum	[202]
Benzoic acid + Essential Oil compounds	CRINA^®^ (Benzoic acid + Thymol, eugenol, and piperine) Poultry Plus	Metabolites and Essential Oil	**EA:** reduce LS	[203]
Synbiotic + Essential oil	*B. licheniformis* + mannan-oligosaccharides and β-glucans + capsaicin (chili peppers) + curcuma (from turmeric)	Probiotic + prebiotic combination + Essential Oils	**NE model (Mix** ***Eimeria*** **spp. +** ***Clostridium perfringens*****):** reduced mortality (0–14 D), improved ADG, reduced LS in duodenum, upregulate mRNA CLDN-3, reduced IFN-γ, IL-10 and sIgA	[204]

BW, body weight; BWG, body weight gain; CAT-1, cationic amino acid transporter-1; CLDN, claudin; FCR, feed conversion ratio; EA, *Eimeria acervulina*; EM, *E. Maxima*; ET, *E. Tenella*; HDP, host defense peptides; IL, interleukin; iNOS, inducible nitric oxide synthase; LS, lesion score; MDA, malondialdehyde; NE, necrotic enteritis; NO, nitric oxide; OPG, oocyst per gram; SOD, superoxide dismutase; ZO-1, zonula occludens-1.

## Data Availability

All the data presented in this study are available in this article.

## Abbreviations

The following abbreviations are used in this manuscript:

ACI	Anticoccidial Index
BCFA	Branched-Chain Fatty Acid
BW	Body Weight
BWG	Body Weight Gain
CAT-1	Cationic Amino Acid Transporter-1
CLDN-1	Claudin-1
FCR	Feed Conversion Ratio
EA	*Eimeria acervulina*
EM	*Eimeria maxima*
E. mi	*Eimeria mitis*
EN	*Eimeria necatrix*
ET	*Eimeria tenella*
IL	Interleukin
LS	Lesion Score
NE	Necrotic Enteritis
NO	Nitric Oxide
OPG	Oocyst Per Gram
ZO-1	Zonula Occludens 1
